# Combinatorial Gene Regulation Using Auto-Regulation

**DOI:** 10.1371/journal.pcbi.1000813

**Published:** 2010-06-10

**Authors:** Rutger Hermsen, Bas Ursem, Pieter Rein ten Wolde

**Affiliations:** 1 Center for Theoretical Biological Physics, University of California, San Diego, California, United States of America; 2 FOM Institute AMOLF, Amsterdam, The Netherlands; University of Tokyo, Japan

## Abstract

As many as 59% of the transcription factors in *Escherichia coli* regulate the transcription rate of their own genes. This suggests that auto-regulation has one or more important functions. Here, one possible function is studied. Often the transcription rate of an auto-regulator is also controlled by additional transcription factors. In these cases, the way the expression of the auto-regulator responds to changes in the concentrations of the “input” regulators (the response function) is obviously affected by the auto-regulation. We suggest that, conversely, auto-regulation may be used to optimize this response function. To test this hypothesis, we use an evolutionary algorithm and a chemical–physical model of transcription regulation to design model *cis*-regulatory constructs with predefined response functions. In these simulations, auto-regulation can evolve if this provides a functional benefit. When selecting for a series of elementary response functions—Boolean logic gates and linear responses—the *cis*-regulatory regions resulting from the simulations indeed often exploit auto-regulation. Surprisingly, the resulting constructs use auto-activation rather than auto-repression. Several design principles show up repeatedly in the simulation results. They demonstrate how auto-activation can be used to generate sharp, switch-like activation and repression circuits and how linearly decreasing response functions can be obtained. Auto-repression, on the other hand, resulted only when a high response speed or a suppression of intrinsic noise was also selected for. The results suggest that, while auto-repression may primarily be valuable to improve the dynamical properties of regulatory circuits, auto-activation is likely to evolve even when selection acts on the shape of response function only.

## Author Summary

Bacteria adjust which proteins they make, and how many copies of each kind, depending on their environment. The production rate of each regulated protein is controlled by a special class of proteins called transcription factors. The rate at which a certain protein is produced usually depends on the cellular concentrations of a few such transcription factors. When circumstances change, the concentrations of these transcription factors alter too and consequently the production rates of all proteins regulated by them are adjusted. Interestingly, many transcription factors also regulate their own synthesis rate. This suggests that this self-regulation must have one or more important functions. In this article we study one possible function. In order for cells to function properly each protein concentration has to respond in a particular way to changes in transcription factor concentrations. We have studied how bacteria can optimize and fine-tune these responses. To this end, we formulated a physical model of the regulation by transcription factors and performed computer simulations. These simulations show that self-regulation—and in particular self-activation—is often a useful tool to achieve the prescribed response. Therefore we conclude that natural selection on the regulation of protein levels could naturally lead to self-regulation.

## Introduction

Many transcription factors (TFs) in *Escherichia coli* regulate the transcription rate of their own gene. In fact, 59% of the TFs are known to auto-regulate and the list is growing [1], [2]. Negative auto-regulation (auto-repression) occurs more frequently than positive auto-regulation (auto-activation), but both are very common: 71 auto-repressors and 34 auto-activators are found in the current databases (including 9 TFs that have binding sites for auto-activation as well as for auto-repression). This suggests that auto-regulation has one or several important functions [3], [4]. In this paper, one possible function is explored. In general, the expression level of a gene is a function of the concentrations of the TFs that regulate its transcription rate. We propose that auto-regulation can naturally be used to optimize the shape of this response function.

Auto-regulating transcription factors are typically regulated by other TFs too. In fact, 23 auto-regulating TFs in *E. coli* are known to respond to at least *two* additional regulators [1], [2]. In such cases, the response of the regulated TF to changes in the “input” TF concentrations must reflect an interplay between regulation and auto-regulation. Conversely, this suggests that auto-regulation could emerge as a result of natural selection on the shape of these responses.

In the past years, several other functions of auto-regulation have been proposed. Negative auto-regulation has been shown to decrease the sensitivity of expression levels to intrinsic fluctuations in the transcription rate under certain conditions [5]–[7] and to mitigate variations due to changes in the bacterial growth rate [8]. In addition, auto-repression can speed up the response of expression levels after a sudden change in conditions [9], [10]. In the presence of time delays, it can also create oscillations [11]. On the flip side, negative auto-regulation tends to reduce the sensitivity of the expression level to input signals [12], [13]. The effects of positive auto-regulation are usually opposite to those of auto-repression: it slows down responses and tends to amplify intrinsic fluctuations. At first sight, these qualities may not seem very desirable. Yet, a slow response can be beneficial if a sensitive response to persisting signals is desired while fast fluctuations in the input signal should be ignored [12]. Occasionally the fact that auto-activation can provide bi-stability may also be useful [14]. Each of these qualities could be relevant in some cases; our new suggestion does not contradict or replace any of them.

To study the benefits of auto-regulation we use a computational approach that we developed recently [15]. In this approach, an evolutionary algorithm and a physical–chemical model of transcription regulation are integrated to design *in silico cis*-regulatory regions with predefined response functions. The evolutionary algorithm subjects a population of model *cis*-regulatory regions to rounds of mutation and selection. The mutations are introduced at the level of base-pair sequences while the selection step is based on the emerging network properties calculated using the model of transcription regulation. In the course of these simulations complex promoter designs develop that perform the desired function; these designs often reveal new design principles. In earlier work, auto-regulation was not included in this method. In contrast, we now use an extended version of the method to design *cis*-regulatory constructs that *can* exploit feedback.

Many *cis*-regulatory regions in real cells essentially implement logical decisions [15]–[17]. We therefore study the class of response functions that can be interpreted as analogue equivalents of logic gates. *Gates* are computational devices that produce an output signal depending on one or more input signals; *logic gates* are gates that implement a binary (Boolean) decision rule. For example, a transcriptional AND gate would be a gene whose expression (the “output”) is regulated by two TFs (the “inputs”, TF1 and TF2) such that it is transcribed only if both TF1 *and* TF2 have a sufficient expression level [16]. We refer to Table 1 for the definitions of other logic gates. Even though it has proven fruitful to think of promoters as analog approximations of logic gates, we stress that gene expression levels are of course not actually binary and that we do not treat them as binary in the models below.

**Table 1:** Truth tables of transcriptional logic gates.

input TFs		output ( 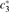 )					
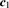	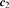	and	or	nor	nand	act †	IN†
low	low	low	low	high	high	low	high
low	high	low	high	low	high	low	high
high	low	low	high	low	high	high	low
high	high	high	high	low	low	high	low

**†:** The act and inh gates have only one input; they depend on 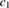 only.

Logic gates are devices that perform elementary binary computations, mapping multiple input signals to one output signal. Here we consider *transcriptional* logic gates. Transcription systems are analogue systems and therefore never truly binary, but it is often useful to think of them as continuous approximations of logic gates. Here, we consider logic gates with one or two inputs (the concentrations 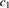 and 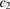 of two transcription factors), and one output (the steady-state expression level of the regulated gene: 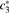). The table specifies, for the six logic gates used in our study, the value of the appropriate output 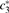 (“low” or “high”) for all input concentrations 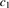 and 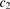 (“low” or “high”). All gates are defined for input concentrations in the domain 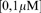 only; concentrations above 500nM are considered high and those below 500nM are low. The acronyms of the gates summarize their function; for instance, the output of an AND gate should be high only when both 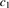 *and* 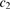 are high. Note that that by definition the ACT (activate) and IN (inhibit) gates have only one input, 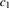; indeed, their output does not depend on 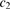.

We analyze the *cis*-regulatory sequences resulting from the simulations by calculating DNA footprints for the resulting transcription factors and promoter sequences. These footprints show that auto-regulation—in particular auto-*activation*—is often used in these *cis*-regulatory regions; indeed, further analysis shows that auto-activation can be used to construct “better” transcriptional logic gates by allowing for more switch-like, “steep” response functions. However, the use of auto-regulation in shaping response functions is not limited to creating switch-like functions. To demonstrate this, we also applied our method to the design of *cis*-regulatory constructs that respond in a *linear* fashion to input concentrations. Again we find that auto-activation emerges spontaneously in the results.

Finally, we also performed simulations in which we selected for designs with desirable dynamical qualities. First, we adjusted the method to select for gates with a short response time. Second, we performed selection against intrinsic fluctuations. In agreement with earlier results [5]–[7], [9], [10], auto-repression evolved in both cases, demonstrating how auto-repression can be used to speed up response times or to reduce intrinsic fluctuations.

Before describing the results we first provide a detailed description of the model and the algorithm used.

## Methods

We combine a model of transcription regulation and an evolutionary algorithm to design *in silico cis*-regulatory regions with a predefined function. The model of transcription regulation is constructed such that all properties of a model regulatory network follow entirely from the sequences of TFs and *cis*-regulatory regions. Binding sites of TFs are therefore not specified beforehand but appear gradually in the course of the simulations. The model is an extension to the one described in detail in our earlier publication [15]. The main innovation is that auto-regulatory interactions are now also included, so that auto-regulation can evolve if this is beneficial.

### Model of transcriptional regulation

**Figure 1:**
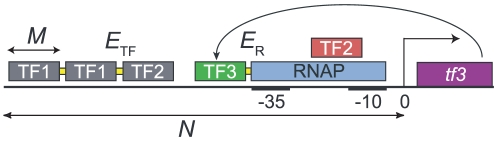
Illustration of the model of transcription regulation. The model describes the transcriptional regulation of a gene *tf3* by two transcription factors, TF1 and TF2. In addition, auto-regulation is included: the gene product TF3 of *tf3* can regulate the transcription of *tf3*. TFs act by binding to *tf3*'s *cis*-regulatory region, represented as a string of 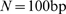 located directly upstream of the start of transcription. The TF binding domains count 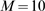 amino acids, and bind to binding sites of length 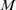 bp. TFs can bind anywhere on the *cis*-regulatory region but with varying affinity determined by the DNA sequence. When two TFs bind within a distance less than 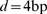, they interact with energy 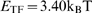, as is indicated schematically by a yellow connection between the TFs. This way cooperative binding is included. The core promoter, consisting of the 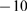 and 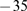 hexamers, is marked; when RNA polymerase (RNAP) binds to it, it blocks both hexamers and the spacer between them. When a TF binds close to the RNAP we assume an interaction energy 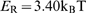; thus the mechanism of regulated recruitment is included. The TF that binds to a site overlapping with the core promoter is red to indicate that it represses transcription by steric hindrance; the green TF is an activator, as it recruits RNAP from its binding site. The gray TFs bind too far upstream to influence the transcription rate.

We consider one “output” gene, *tf3*, and at most two “input” transcription factors, TF1 and TF2. The gene *tf3* codes for another transcription factor called TF3. All three TFs can regulate the transcription rate of *tf3* by binding to its *cis*-regulatory region. (See Fig. 1 for an illustration of the model.)

The *cis*-regulatory region and the TFs are represented as nucleotide sequences and amino-acid sequences respectively. All TFs can bind anywhere on the *cis*-regulatory region, but the affinity of a TF for a particular site depends on the sequences of the TF and the site. For our purpose, it is sufficient to only model the DNA-binding domains of the TFs explicitly. We assume that 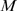 amino-acids in these domains are responsible for the DNA-binding specificity and therefore represent each TF as an amino-acid sequence of length 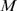. We choose 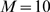 in our simulations because known binding sites in *E. coli* typically have length 6 to 15 and usually one base pair interacts with 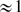 amino acid in TF–DNA binding [2]. The *cis*-regulatory region of *tf3* is a base-pair sequence of length 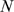; we take 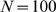 because in *E. coli* most transcription factors bind within 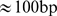 from the start of transcription [18]. By the rules specified below all interactions between TFs, RNA polymerase (RNAP) and the *cis*-regulatory region can be deduced from these sequences; therefore each transcriptional gate is completely specified by them.

The various molecules interact in the following ways:

#### TF–DNA interactions

Each TF 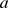 can bind to any site 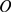 on the *cis*-regulatory region, but the affinity of 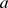 for 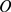 depends on the DNA sequence of 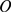 and the amino-acid sequence of 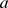. Each amino acid interacts with exactly one base pair, and the total binding free energy 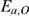 is the sum of the contributions of each amino-acid–base-pair contact. This means that the binding free energy of a TF with amino acids 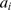 to a binding site with base pairs 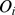 is given by
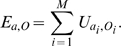
(1)Here 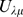 is a 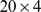 matrix containing the binding free energies associated with each amino-acid–base-pair contact. We used the matrix given in [19], based on christallographically solved protein–DNA complexes.

#### TF–TF interactions

TFs are assumed to mutually interact in two ways. First, TFs cannot bind simultaneously to overlapping binding sites due to steric hindrance. This introduces competition for binding to overlapping binding sites. Second, if two TFs bind within a distance of 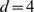 base pairs, they interact cooperatively: if one of the TFs is bound, the equilibrium dissociation constant of the other TF is decreased by a factor 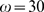, corresponding to an interaction energy 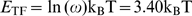. In reality, 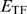 is typically 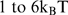 (about 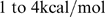), leading to 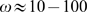 [20]. Many TFs interact by direct contact, but indirect interactions may also occur, for instance if one TF bends, stretches or super-coils the local DNA and thus increases the affinity of the other TF. In the context of this model it is irrelevant which mechanism is responsible for the cooperativity.

#### RNAP–DNA interactions

RNAP is assumed to bind only to the basal promoter. The affinity of the 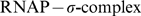 for the core promoter is determined by the 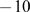 and 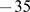 consensus hexamer sequences on the *cis*-regulatory region of *tf3*. We determine the binding free energy of 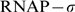 for a core promoter by comparing the 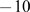 and 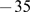 hexamers to a large set of real *E. coli* promoters, taken from reference [21]. To every base pair 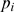 at position 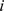 within the 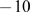 and 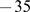 hexamers we assign a score 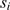; it equals the fraction of real *E. coli* promoters that have 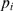 at that particular position, normalized by the random fraction 1/4. Next, the binding energy 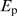 of RNAP to that particular core promoter can be estimated by [22]–[24]:
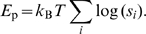
(2)Thus, promoter sequences that are more similar to the consensus sequence of real promoters yield a higher affinity.

#### TF–RNAP interactions

TFs interact with RNAP and influence the transcription rate by the principles of regulated recruitment [25]. If TFs bind to a site overlapping the basal promoter they block the binding of RNAP and thus repress transcription. On the other hand, if they bind within a distance 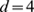 from the promoter we assume that the TF and RNAP interact cooperatively. If the TF is bound to its binding site, this decreases the dissociation constant of RNAP binding to the promoter by a factor 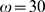 (corresponding to an interaction energy 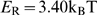); thus, the TF recruits RNAP and activates transcription.

The transcription rate of *tf3* is assumed to be proportional to the equilibrium fraction of time the promoter is occupied by RNAP [20]. This occupancy can be computed given all sequences and the concentrations of the TFs (denoted by 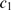, 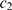 and 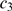). For this we use the statistical mechanics formalism originally developed in Ref. [20] and explained in detail in references [26] and [15]. Briefly, the method calculates the partition sum 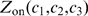 of all states of the system in which RNAP is bound to the promoter, and the partition sum 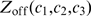 of all states in which RNAP is *not* bound; the fractional occupancy of the promoter is then given by:
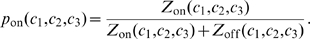
(3)Note that, in general, 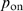 is a function of all three TF concentrations. To efficiently take into account all possible configurations in which TFs could be bound to the *cis*-regulatory region we use the efficient recursive (dynamic programming) algorithm presented in [15].

The model described above contains the generic mechanisms that are responsible for many of the known transcription regulatory interactions. Yet, several mechanisms that play a role in specific systems are not included; for instance, the model does not include DNA looping, extended 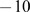 regions or twisting or bending of the DNA by TFs. This suggests that real transcriptional regulatory systems are probably more flexible than our model systems. However, by endowing our model with only the basic features known to be important in virtually any transcription system we ensure that the resulting designs do not rely on exotic mechanisms.

### Model of the dynamics

We model the dynamics of the concentration of TF3, 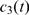, by the following ordinary differential equation:
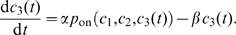
(4)Here 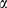 is the maximal production rate of TF3, and 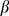 is the degradation rate constant of TF3. The function 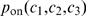 was defined above. In this simplified description, transcription and translation are concatenated and translational regulation is not included.

The concentrations 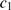 and 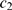 of TF1 and TF2 are considered the inputs of the gate. Assuming that the system is mono-stable (bi-stability is discussed below) equation 4 defines a unique steady state for each set of input concentrations 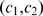 in which 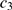 has a value 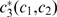. This steady-state concentration is considered the output of the gate. Because time delays between transcription initiation and translation are ignored, oscillations are excluded and 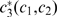 can be calculated by propagating the dynamics numerically from any initial condition until the steady state is reached. (If a gate has only one input, the dependence on 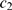 is simply dropped.)

We choose the constants 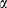 and 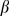 such that 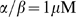; this ensures that 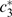 stays within the range 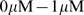. Apart from this ratio the values of 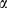 and 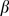 are irrelevant because in this work we are not interested in absolute time scales of the dynamics; for simplicity we use a time unit 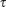 such that 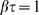.

### Evolutionary algorithm

In order to design networks with a prescribed function an evolutionary algorithm was used. A population of 200 transcriptional gates was subjected to 1000 cycles of mutation, selection and replication. Initially, all gates had random sequences. Auto-regulation was not imposed, but the system was free to exploit it by evolving binding sites for TF3. The details of the evolutionary algorithm were chosen to combine an effective optimization of the gates with computational efficiency; we emphasize that we do not intend to faithfully mimic biological evolution.

Several types of mutations were included. First, a base substitution could occur in *cis*-regulatory sequences (with probability 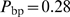 per *cis*-regulatory region). If this happened, a base pair was selected at random from the *cis*-regulatory sequence and substituted by a randomly chosen nucleotide. Second, insertions or deletions of a random base pair occurred in *cis*-regulatory regions (with probability 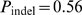). Third, we applied point mutations to the sequences of the TFs (with probability 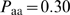 per TF), in which case one randomly chosen amino acid in the sequence was replaced by a random alternative. The exact mutation rates are not crucial for the results, as long as the rates are (i) high enough to generate significant variation and (ii) low enough to allow high-quality gates to persist in the population.

**Figure 2:**
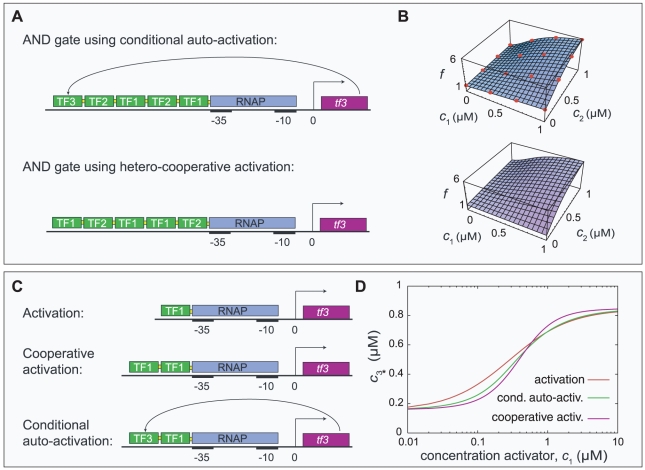
Conditional auto-activation. In some simulation results, auto-activation occurs only in the presence of another transcription factor (TF). We call this conditional auto-activation. The figure presents two examples. Fig. A: The promoter of an AND gate using conditional auto-activation and, for comparison, one using hetero-cooperative activation. Both designs emerged in the simulations. In the first case, the regulated gene *tf3* codes for a transcription factor TF3 that binds to its own *cis*-regulatory region. However, from its binding site, TF3 can activate transcription only indirectly, by facilitating the binding of TF1 and TF2 to their binding sites. As a result, the auto-activation depends on the presence of TF1 and TF2 (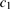 and 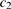). Fig. B shows plots of the expression level of *tf3* (fold-change 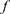 vs. the concentrations 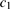 and 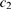 of TF1 and TF2) resulting from the *cis*-regulatory regions in Fig. A. The red dots show the values of 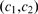 that were used to evaluate the fitness of the gate (see Methods). Fig. C and D show the same mechanism in a simplified model inspired by the simulation results. Plot D compares the response functions corresponding to three activation systems depicted in C. In all cases, a single TF activates the expression of a gene *tf3* coding for another transcription factor, TF3. The first two scenarios constitute conventional activation systems with one or two binding sites. In the third scenario, one binding site is replaced by an operator for TF3, which introduces a positive feedback loop depending on the presence TF1. The binding affinities of all sites are optimized using the fitness function described in the main text. The response of the conditional auto-activation system is clearly sharper/more sensitive than the one using a single activation site. Cooperative auto-activation by two sites, however, leads to a slightly sharper response. The results suggest that conditional auto-activation is an alternative design principle that can be used to sharpen responses.

For the selection step a fitness score 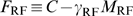 was used. Here 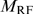 (where RF stands for Response Function) measures the deviation of the response function from a predefined goal function (the desired response function). It was computed as follows. When evaluating gates with two inputs, 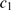 and 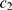, the output level 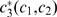 was computed for 16 combinations of the input concentrations: 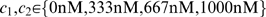 (see the red dots in Fig. 2B). Next, the differences between these output levels and the goal function were computed. 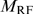 was defined as the sum of the squares of these deviations. If the gate had only one input, the definition was analogous, except that seven input values were used, equally spaced in the interval 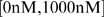. The constant 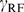 is required to make 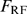 dimensionless (for simplicity, 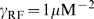) and 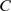 is an arbitrary constant large enough to ensure 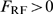. Based on the fitness scores, the top 20% of the population were selected and the remaining gates discarded. Subsequently the population was brought back to its initial size by duplicating gates randomly chosen from the survivors of the selection process.

If auto-activation evolved, the system could become bi-stable. In bi-stable systems, given the inputs 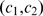 two different values of 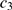 are stable under the dynamics of the system (equation 4) so that the output concentration 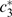 is not uniquely defined by the input concentrations. Even though bi-stability is likely to occur in some real transcription networks we decided that such systems do not qualify as gates, since gates by definition map input states to a uniquely defined output state. The fitness function therefore contained an additional term that was designed to penalize bi-stability. When evaluating the fitness of a gate, we always computed the steady-state value 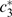 *twice* for 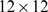 input values 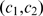: once by propagating the differential equation 4 using initial condition 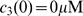 and once using 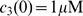. If the results were different, the difference squared was added to the fitness function, which was sufficient to assure that the particular gate was eliminated by the selection process. However, because this method did not exclude bi-stability for all possible input values we also checked afterward whether the results were bi-stable.

### Definition of the gates

All gates are defined for input concentrations in the domain 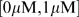 only. The logic gates are specified in Table 1. In addition we define LACT, LIN, MEAN and NMEAN gates. A LACT (linear activate) gate has one input, 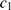, and the output 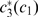 responds as 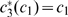. A LIN (linear inhibit) gate also has one input, but responds according to 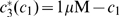. A MEAN gate is linear in two inputs, 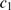 and 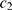, and obeys 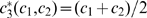. Lastly, we define NMEAN to have the following linearly decreasing response function: 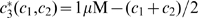.

We repeated the simulations for each of the gates 20 times with different random seeds.

### Quantifying the degree of auto-regulation

In order to quantify the importance of auto-regulation in a particular design we defined the measure 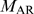 (referred to as the “feedback measure”). First, we calculated the response function 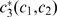 for the particular design. Then we artificially removed all possible binding sites for TF3 by setting the affinity of TF3 for all sites on the *cis*-regulatory region to zero and calculated the response function again; we call the result 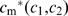. In the absence of auto-regulation one should find 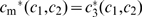, but if auto-regulation does play a role the two functions differ. Therefore the difference between these functions is a measure of the degree of auto-regulation; we define 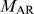 as the mean of the squared differences 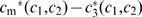 over 16 combinations of the input concentrations 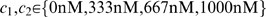 (again, the red dots in Fig. 2). If auto-regulation is not being exploited by a certain design 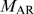 is generally small (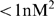). If, on the other hand, auto-regulation is used the resulting value is typically in the range 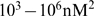. (For instance, if all 16 points shift by 100nM when auto-regulation is removed 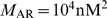.)

### Recognizing binding sites

During the simulations, binding sites emerge in the initially random *cis*-regulatory promoter sequences. However, since the equilibrium binding constants have continuous values there is no fundamental distinction between binding sites and non-binding sites. Recognizing binding sites is further complicated by the fact that, in particular in the presence of cooperativity, weak binding sites can be important. Nevertheless, in order to understand the design principles of a particular gate we wish to identify which binding sites are necessary and sufficient to explain the observed promoter response. This problem is not an artifact of our models: the exact same conceptual problems occur whenever one tries to identify the binding sites of real TFs by experiment.

Since a direct cutoff in terms of the equilibrium constants would eliminate possibly important weak sites we use computational “DNA footprints” (analogous to experimental techniques such as DNase I footprinting) to select those sites that are likely to be important. For each TF 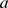 and each site 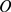, we calculate the steady-state occupancy 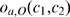 for four sets of input concentrations 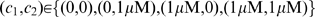. Sites that influence the response of the gate should have a significant occupancy in at least one of these digital footprints.

We define 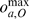 to be the maximal occupancy of site 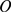 by TF 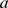 over the four conditions. Figure 4 in Text S1 shows a histogram of these occupancies for all TFs and all sites using data gathered from the results of 200 simulations. This histogram is bi-modal. The vast majority of the maximal occupancies 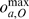 have negligible values but a second peak occurs at 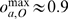. This peak is the result of selection pressure and is associated with functional binding sites. Based on this histogram, we use a rather stringent cut-off at 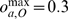 to separate binding sites from pseudo binding sites. Simplified models that only take into account these selected binding sites and assume that all other binding affinities are zero usually accurately reproduce the response function of the full, unsimplified system. In rare cases where this is not the case the threshold can be lowered to obtain more accurate but more complex models; this was not necessary for the examples presented below. (See the Text S1 for more details and examples of footprinting profiles.)

## Results

Below, we describe the results of the simulations. We regularly compare the results to our previous work in which auto-regulation was excluded (Ref. [15]). In some cases the simulations presented here resulted in the same designs as before. But in other cases the designs exploited auto-regulation to arrive at novel and often superior designs. We identified several mechanisms that are used repeatedly in the results and present them below. Details about several of the analyses below can be found in Text S1.

### Conditional auto-activation leads to steep responses

The first mechanism that our scheme elucidated, we called *conditional auto-activation*. This mechanism occurred in AND and ACT (activation) gates (see Table 1 for the definitions), in which cooperative activation plays a key role. In those gates, conditional auto-activation is used to create a steep, switch-like response. As an example, we first discuss the design of AND gates.

In simulations in which auto-regulation was excluded by the method, the resulting AND gate designs always consist of a tandem array of binding sites to which TF1 and TF2 bind cooperatively (see Fig. 2A) [15], [16]. We called this a hetero-cooperative module. This design functions as follows. Crucially, the binding site from which RNAP is recruited (the site directly next to the core promoter) is too weak to considerably activate transcription on its own. As a result, only when TF1 and TF2 are both present at sufficient concentrations they bind cooperatively and activate transcription, as the definition of an AND gate requires. In the new simulations, in which auto-regulation can evolve, this design still emerged in 14 out of 20 simulation runs. Each of these gates has a feedback measure 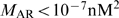, proving that auto-regulation does not play any role.

The remaining 6 simulation runs resulted in conditional auto-activation. In these gates the feedback measure 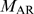 was high, in the range 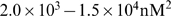. The new design looks very similar to the old one (see Fig. 2A and B). However, the hetero-cooperative module now also contains a binding site for TF3, which leads to a positive feedback loop. Importantly, TF3 bound at its binding site cannot recruit RNAP directly; instead, it interacts with the hetero-cooperative activation module. As a result, the auto-activation is conditional on the presence of TF1 and TF2. As the concentrations of TF1 and/or TF2 increase, the auto-activation is gradually turned on, leading to a sudden (steep, switch-like) response.

The exact same mechanism is exploited by some ACT gates. Out of the 20 simulations of ACT gates, 3 resulted in conditional auto-activation (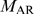 values were 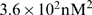, 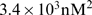 and 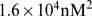), while the other 17 did not use auto-regulation (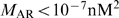).

The basic mechanism can be studied in minimal models inspired by the simulation results. In Fig. 2C and D, we compare three activation mechanisms. The first scenario is conventional activation by a single TF1 binding site. In the second scenario only a homo-cooperative activation module is present, consisting of two binding sites for TF1. In the third scenario, the auxiliary TF1 site is replaced by a binding site for TF3, introducing conditional auto-activation. In all designs we chose the binding site affinities such that they maximize the fitness function for the ACT gate. Conditional auto-activation indeed produces a response that is steeper than the one resulting from the design with a single activator binding site (Fig. 2D). However, the conventional cooperative design with two binding sites gives an even steeper result. The results imply that, after one binding site has evolved for the activator TF1, the response can be improved in two ways: by adding an additional site for TF1 (leading to cooperative activation) or by adding a site for TF3 (resulting in conditional auto-activation). Which design emerges therefore depends critically on the actual sequences and mutations occurring in the population. This explains why cooperative activation and conditional auto-activation show up as alternatives in the simulations.

The effect of conditional auto-activation can be understood quantitatively by studying the minimal model mathematically. The response function 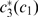 for the minimal model follows from the condition 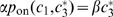 and is given by
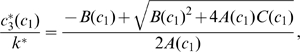
(5)with
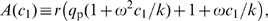


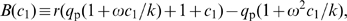


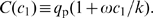
Here 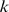 and 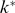 denote the dissociation constants for TF1 and TF3 binding to their respective operator, 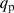 is the concentration of RNAP normalized by the dissociation constant of RNAP binding to the promoter, and 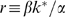. We first consider the limit of 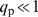 and 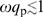. In this limit 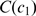 is small as long as 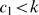. The numerator of 5 can then be approximated by 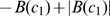. As a result we can distinguish two regimes depending on the sign of 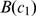:
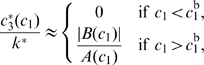
(6)where 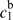 is the border between the two regimes, implicitly given by 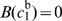:
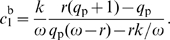
(7)Note that 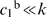 provided 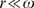; under this condition approximation 6 holds around the transition 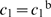. As both 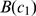 and 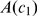 are linear functions of 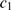, the second regime has the form of a Hill function with Hill coefficient 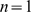. Therefore equation 6 shows that equation 5 behaves like a sharp threshold response. This threshold effect is responsible for the increased steepness of the response due to conditional auto-activation.

The maximal expression following from equation 5, at full activation, is 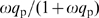. This demonstrates that in the limit of 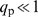 the maximum expression level becomes very low (for a given value of 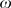). On the other hand, if 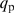 is increased the term 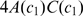 becomes more and more significant and the transition between the two regimes in equation 6 becomes more and more gradual. Consequently, in the optimized functions plotted in Fig. 2 the values of 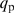 reflect a compromise between the opposing requirements of having a high maximal expression (requiring a large 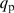) and a sharp threshold response (requiring a small 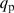).

The steepness of a function 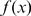 in the point 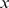 can be formalized by the *sensitivity*, defined by 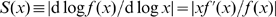. The sensitivity of a Hill function is limited by the Hill coefficient 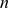, which is equal to the number of cooperatively interacting binding sites for the input TF. We therefore ask if a similar limitation applies to the minimal model of conditional auto-activation. From equation 5 the sensitivity function 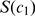 can be derived straightforwardly. The result is rather cumbersome and therefore an exact expression for the *maximal* sensitivity is hard to obtain. However, since the most sensitive part of the function is in the region where 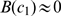 (*i.e.*, close to 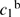), the maximal sensitivity can be approximated by 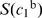. In the limit of large 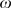 this expression converges to
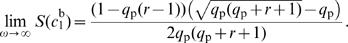
(8)Importantly, since we evaluated the sensitivity in a point close to but not exactly at the maximum, this approximate result is a conservative estimate: the true maximum cannot be lower than this. In the limit of small 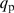 the maximum sensitivity diverges as 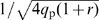, which proves that the sensitivity of response function 5 does not have a theoretical upper limit, unlike those of Hill functions.

So far we have neglected the dynamical properties of the designs because the current model only considers the steady-state response of the system. As we mentioned, auto-activation tends to slow down the response time of the system. Therefore, in systems where the speed of response is of great importance cooperative regulation is expected to outperform conditional auto-activation. Selection on response speed is discussed in more detail below.

### Auto-activation can sharpen repression

A second feedback pattern emerges in logic gates in which repression is important, notably the NAND, NOR and IN (inhibition) gates. As it turns out, whenever steep repression is required, we also find strong auto-activation; this occurred in every simulation run for our NAND, NOR and IN gates (20 repeats each), with 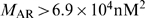 in all cases. We present the NAND gate as an example.

**Figure 3:**
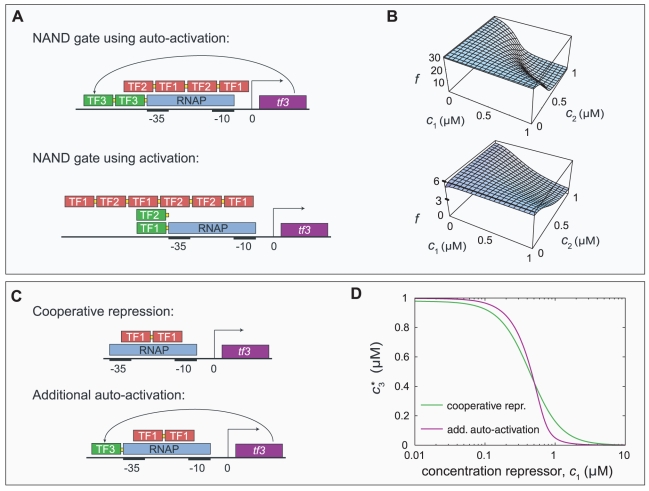
Sharp and complete repression using auto-activation. In the simulations, auto-activation evolved in every gate that requires strong repression. This figure shows two examples in which auto-activation indeed aids sharp and thorough repression. Fig. A and B depict NAND gates resulting from the simulations. When auto-regulation is not allowed by the method, the input TFs have both activating and repressing binding sites, as reported earlier [15] (in Fig. A and C, red boxes represent repressor sites and green boxes activator sites). When auto-regulation is included (*i.e.*, the regulated gene *tf3* codes for a transcription factor TF3 that can bind to its own *cis*-regulatory region) auto-activation emerges. Gene *tf3* is still repressed by a hetero-cooperative module consisting of binding sites for TF1 and TF2. At low concentrations of TF1 and TF2 the auto-activation counteracts the repression module; as a consequence, the response to the concentrations of TF1 and TF2 is very sharp and the fold-change 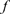 high, as can be seen in Fig. B. In Fig. C and D we study the mechanism in a simpler model system. Fig. C shows the *cis*-regulatory regions of two slightly different repression systems. In both cases, a transcription factor TF1 represses a gene *tf3*, coding for a second transcription factor TF3, by binding cooperatively to a pair of repressor sites. In the second scenario, an auto-activation site for TF3 is present as well. Fig. D presents the steady-state expression level of *tf3* as a function of the repressor concentration. In both alternatives we optimized the binding sites using the fitness function described in the main text. Clearly, the second scenario leads to a more sensitive repression curve than the first. The presence of auto-activation allows for stronger repressor sites; consequently, as the concentration of TF1 increases the displacement of RNAP from the promoter by the repressor is more effective (*i.e.*, the remaining expression level at 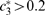 is much lower than in the cooperative repression case).

Fig. 3A shows the *cis*-regulatory region of a typical NAND gate using auto-activation. The corresponding response function plotted in Fig. 3B indeed shows an excellent NAND-like behavior. As quantified below, in fact it performs better than the design without auto-activation reported earlier and reproduced for comparison in Fig. 3A and B [15].

The design that resulted when auto-activation was excluded is composed of a hetero-cooperative repression module (a tandem series of repressor sites to which both input TFs bind cooperatively). The function of this module is to repress transcription only when both TF1 and TF2 are present in sufficiently high concentrations, as required of a NAND gate. In Ref. [15] we pointed out that in the simulation results such a repression module was always accompanied by strong *activation* sites for both input TFs. This counter-intuitive feature turned out to enhance the sharpness of the response. At low TF concentrations, the activation sites counter-act the repression module, so that the expression stays high. At higher TF concentrations, however, the repression module dominates and represses transcription. Under the parameters used designs of this type reached a modest fold-change of 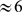 and a deviation measure 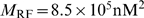 (see section “Evolutionary algorithm” in the methods section for the definition of 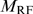).

In the new results (Fig. 3) the activation sites for TF1 and TF2 have disappeared, but instead we find auto-activation. In the absence of TF1 and TF2, *tf3* is highly expressed, aided by auto-activation. As the concentrations of TF1 and TF2 are increased, the repression module starts to compete with the auto-activation module. Quite suddenly, the repression module wins this competition and displaces RNAP from the promoter. The strong, cooperative repression module now leads to a rather complete inhibition. The new design can lead to fold-changes of 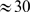 and a deviation measure of 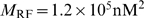.

To study the mechanism responsible for the steepness of the response function we again analyzed a minimal model. In Fig. 3C and D two scenarios for an IN gate are compared. In the first scenario, a transcription factor TF1 cooperatively binds to a pair of repressor sites to inhibit the gene *tf3*. In the second scenario we use the same configuration, but add an activator site for TF3. Thus, auto-activation competes with cooperative repression. The fitness of each design is optimized using the fitness function for the IN gate. As can clearly be seen in Fig. 3D the second scenario, using conditional auto-activation, results in a steeper and more complete repression. Figure 1 in Text S1 shows plots of the sensitivity 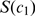 as a function of 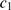 for the response plots in Fig. 3D and clearly demonstrates that auto-activation enhances the sensitivity.

Does the sensitivity of the response function 9 have an upper bound, as is the case for Hill functions? To answer this question we again study the minimal model mathematically.

The response function 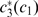 of the minimal model is given by
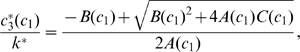
(9)with
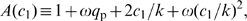
(10)

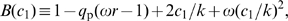
(11)

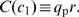
(12)We first describe the limit in which 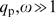 and 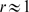. The form of this equation is obviously similar to equation 5 for conditional auto-activation. However, here 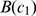 is large and *negative* (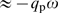) when 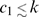. In this regime, 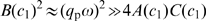, so that the numerator is approximated by 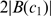. As 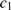 increases, 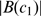 decreases while the denominator increases; therefore the expression is rapidly repressed. This regime ends suddenly as 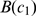 reaches zero, at 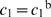; at this point the expression is almost fully repressed.

The sensitivity function 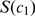 can be derived from equation 9. The exact expression is again too cumbersome to derive the maximal sensitivity analytically. However, the most sensitive region of the response plot is again expected around 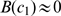 so that we estimate the maximal sensitivity as
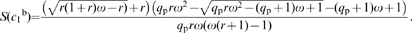
(13)For large 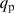 this converges to
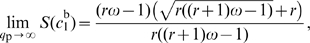
(14)which for large 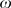 approaches 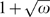. Numerical tests demonstrate that this conservative approximation becomes excellent for 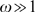 (data not shown). In the absence of auto-regulation, the sensitivity cannot exceed 2, the number of repressor sites. Equation 14 demonstrates that in the presence of auto-regulation the sensitivity can easily exceed 2 but is nevertheless limited given 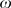.

We note that the sensitivity is optimal for 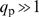. Hence, unlike the case of conditional auto-activation the requirements of a high maximal expression and a high sensitivity do not contradict. In the simulations as well as in reality, however, the promoter strength is bounded by other factors. Clearly the binding affinity of RNAP for the promoter is bounded by the physics of RNAP–DNA binding. A less obvious constraint follows from the fact that the expression switches from high to low around 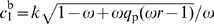; if the repression is to occur at reasonable TF1 concentrations (the simulations impose the interval 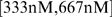) high values of 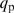 require low values of the dissociation constant 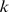 (*i.e.*, strong repression). Finally, a high sensitivity in one point does not guarantee that the response function switches from high to low in a narrow interval as is required by the fitness function; this explains why in the plots in Fig. 3 the maximal sensitivity is not optimal (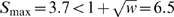).

### Linear repression benefits from auto-activation

In both previous cases, auto-regulation was used to obtain the steep or switch-like behavior required to approximate the binary responses of logic gates. Indeed, sharp responses are observed and probably required in many real examples; nevertheless many genes respond in a more gradual manner to their input signals (Ref. [17] provides examples of both sharp and gradual responses). Is auto-regulation also useful in cases where a gradual response is required? To test this, we now turn to the results of simulations with *linear* goal functions.

**Figure 4:**
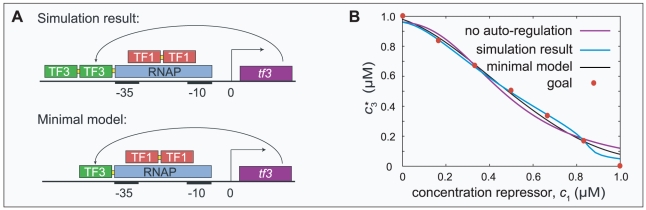
Linear repression using auto-regulation. If in the simulations we selected for a linearly decreasing response function (a LIN gate), auto-activation emerged. The resulting *cis*-regulatory region is schematically depicted in Fig. A. Red boxes and green boxes represent repressor and activator sites, respectively. The corresponding response function is plotted in Fig. B, alongside the results of a simulation in which auto-regulation is excluded. The auto-activation indeed manages to straighten the repression curve. The seven red dots in Fig. B show the goal function that is used in the fitness function: gates are considered better if their response function fits these points better (see Methods). We again studied a simplified model in more detail; the *cis*-regulatory region and response function of this minimal model are also shown. Indeed, the simple model system with appropriate binding site affinities fits the goal points better than the design without auto-regulation.

Indeed, simulation results for linear repression (*i.e.*, the LIN and NMEAN gates) always use auto-regulation, with 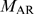 in the range 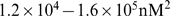. As can be seen in Fig. 4, approximately linear repression can be obtained when repression is combined with auto-activation; the deviation measure for the simulation result shown is 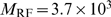. The same figure shows results of simulations in which auto-regulation is excluded. In that case a large cooperative repression module results, which leads to a less linear result (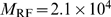).[Fig pcbi-1000813-g005]

**Figure 5 pcbi-1000813-g005:**
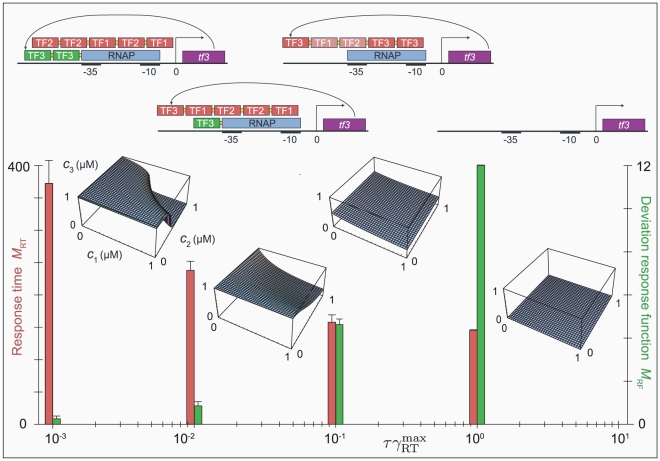
NAND gates at increasing selection pressure on response speed. On the one hand, the sensitivity of the response function of a NAND gate is improved by auto-activation. On the other hand, the response speed of the gate is enhanced by auto-repression. Consequently, if selection acts both on the response function and the response time, the simulation results are a compromise and depend critically on the relative magnitudes of the two selection pressures. The figure shows representative response functions and promoter designs of NAND gates resulting from four values of the parameter 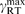, which controls the weight of the response speed in the total fitness function 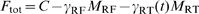. (The irrelevant constant 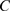 merely serves to ensure that 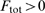.) The average values of the measures 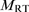 (measuring the response time in arbitrary units 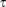) and 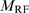 (the deviation of the response function from the goal function in units 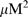) for each condition are also plotted. By definition, low values of 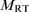 and 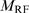 correspond to *good* performance. For the lowest value 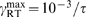 the response function is optimized and shows an excellent NAND gate. Due to strong, cooperative auto-regulation, the response is very sharp and almost bistable in the transition region, but the response speed is low. At 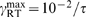 the result is a compromise: the quality of the response function is clearly reduced but the response speed is higher. Still auto-activation evolves, but it is weaker and non-cooperative and combined with weak auto-repressing binding sites. At 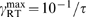 auto-repression fully takes over; the response function is crippled but the response speed is high. If the selection pressure on response time is increased even further (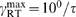) the response speed is fully optimized by disabling the response altogether.

Again, we analyzed the mechanism through a slightly simplified model presented in the same figure. The promoter design of the simplified model is identical to the one presented in Fig. 3C, where it was used to demonstrate how auto-activation can provide sharp responses. In essence, the difference between the two cases is that in the IN gate the two repressor sites have the same affinity, whereas in the LIN case they do not: one of them is many times weaker than the other (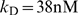 vs. 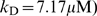. As a result, the repression is introduced gradually as the repressor concentration increases.

Linear repression requires that 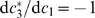 in the domain 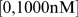. Since 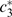 is defined as the solution of 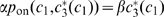, we can take the total derivative of this relation to arrive at
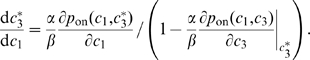
(15)In the absence of auto-regulation the denominator equals 1. In this case 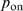 is a Hill-type function of 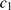 and therefore its derivative is not constant. In the presence of auto-regulation the denominator can be used to correct some of the variation in the numerator. (See Text S1.)

In contrast, in the simulation results for linear *activation* (both the LACT and the MEAN gates) auto-regulation is never used. To test if these results are an artifact of the algorithm, we studied a series of models (see Text S1). Each model is a possible layout of transcription factor binding sites and includes auto-regulatory sites. For each of the models, we optimized the affinities of all binding sites with respect to the fitness score, using a standard Nelder–Mead optimization routine. Consistent with our simulations, in the solutions for all models the affinities of the auto-regulatory sites vanished. Even though the list of models tested is not exhaustive, this suggests that auto-regulation is not helpful in constructing LACT or MEAN gates.

To illustrate an important difference between linear activation and linear repression we provide the following general argument. Suppose that an accurate LACT gate can be constructed using auto-regulation. By definition the response function should then be 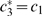 in the interval 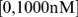. Consequently, 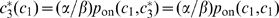 in this interval. Interestingly, this shows that if all TF3 binding sites in the *cis*-regulatory region are replaced by binding sites for TF1—resulting in a gate without auto-regulation—*the exact same response function should be obtained*. Even though this argument does not prove that auto-regulation cannot be used to construct LACT gates, it does show that if a high-quality LACT can be constructed with auto-regulation a similar response can always be obtained without it as well. This is in stark contrast with the linear repression case, where 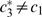.

### Selection for quick responses or against noise leads to auto-repression

Surprisingly, auto-repression does not show up in any of the simulations described so far, whereas auto-activation features regularly. As we mentioned in the introduction, previous studies have shown that negative auto-regulation can be used to diminish intrinsic noise and to speed up response times. In the simulations presented so far, such qualities were not rewarded. Therefore we asked if auto-repression would emerge if we did select for such dynamic properties on top of our usual selection criteria.

First, we used a heuristic measure 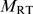 (where RT stands for Response Time) to select for a quick response to changes in the input parameters; it was computed as follows. For 16 combinations of input concentrations 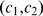 (corresponding to the red dots in Fig. 2) we numerically solved the differential equation 4 with two different initial conditions: 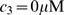 and 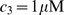. The solutions were used to measure the time it took for the system to approach the steady-state value up to a small distance 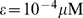. The measure 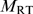 was defined as the sum of all 32 response times. The total fitness function, combining selection on the response function with selection on the response time, was 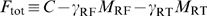, where the factor 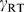 was used to tune the relative strength of the selection on the response time. Again, 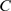 is an irrelevant constant ensuring that 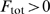.

In fact, the initial condition of the simulations, in which the gate is completely dysfunctional, is a local optimum of this fitness score. This is because initially the steady-state expression level is negligible so that the response time for initial condition 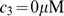 is practically zero. Even though mutations that increase the constitutive promoter activity improve both the response function and the response time for initial condition 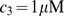, the concomitant increase in the response time for initial condition 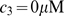 dominates. To ensure that the simulation was not trapped in this local optimum 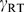 was increased slowly from 0 to 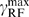 in the course of the simulations, according to:
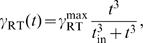
(16)where 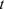 is the simulation time (*i.e.*, the cycle number of the evolutionary algorithm), and 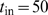.

Second, we selected against intrinsic noise, *i.e.*, fluctuations in the concentration of 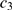 due to the stochasticity of the processes involved in the production and degradation of TF3. This type of noise should be contrasted with extrinsic noise, which here is understood to be the noise due to fluctuations in the input concentrations 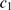 and 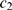 or due to changes in RNAP concentrations [12], [27]. Even though extrinsic noise is generally important too [28], the treatment of extrinsic fluctuations involves subtleties that are beyond the scope of this work, such as the question which changes in 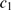 and 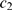 should be considered changes in the input signal and which should be considered noise. We therefore only consider intrinsic noise.

In order to treat intrinsic fluctuations in a tractable manner we now replaced the ordinary differential equation 4 by the following stochastic differential equation:
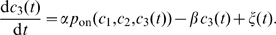
(17)The term 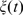 represents Gaussian white noise and is characterized by (see Text S1):
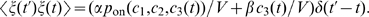
(18)The first term on the right-hand side describes the noise in the production of TF3 while the second term describes the stochasticity in the degradation of TF3. Both terms depend explicitly on the volume 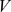 because, at constant concentration, the copy number of TF3 scales with 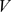 which affects the variance in 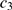.

In Text S1 we show that the standard deviation 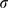 of the concentration 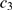 can be approximated as:
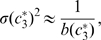
(19)with
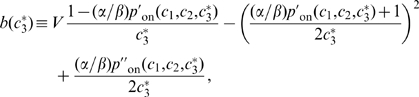
(20)where 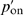 and 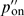 are the first and second partial derivatives of 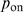 with respect to 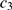, and 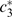 is the steady-state solution of the deterministic equation 4. We computed the right-hand side of equation 19 numerically for 16 input values 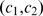 (again, corresponding to the red dots in Fig. 2) and treated the sum of the results as an additional fitness measure 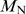 (where N stands for Noise). The strength of selection against noise was again increased gradually during the simulations (analogous to equation 16). The total fitness function thus became 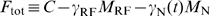.

We performed simulations with several values for 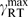 and 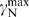: 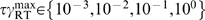 (where 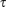 is the arbitrary unit of time, see Methods), and 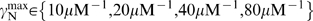. Indeed, in these simulations auto-repression emerged.

In activating gates (ACT, AND, OR) auto-repression resulted in all simulation runs with 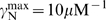 or 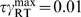. The auto-repression was invariably strong, with 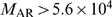, and mediated by multiple cooperative binding sites. If 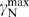 or 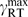 were further increased eventually the resulting *cis*-regulatory regions became completely dysfunctional; this can be understood from the fact that both the response time and the noise reduction can be optimized by abolishing expression altogether. Figure 7 in Text S1 demonstrates how the properties of resulting OR gates changed as a function of 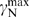. As 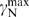 is increased the deviation of the response from the ideal OR gate, measured by 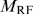, increases, while the noise, measured by 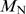, decreases.

As we explained, the response functions of the NAND, NOR and IN gates benefit from auto-activation; in those gates auto-activation occurred unless the selection pressure on the dynamical properties dominated (*i.e.*, if 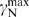 or 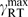 were large), in which case the quality of the response functions was negatively affected. Fig. 4 shows results from simulations selecting for NAND gates at various values of 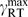. As the selection pressure on response time was increased the response functions became more and more compromised. Auto-activation resulted for 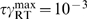 and 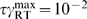; in the former case the promoter designs were of the type shown in Fig. 3A, while in the latter case only one auto-activation site remained. Interestingly, in most of the simulation runs (18 out of 20) at 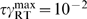 a weak auto-repression site shows up in conjunction with the auto-activation site. These weak auto-repression sites are incorporated in the hetero-cooperative repression module and have a high occupancy only at high concentrations of TF1 and TF2; analogous to conditional auto-activation, this effect could be called conditional auto-repression. At 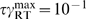 the auto-activation was replaced by strong auto-repression mediated by a single or multiple binding sites and the sites for the input TFs were very weak. Finally, at 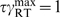 the resulting gates became completely dysfunctional and no significant binding sites remained.

### Auto-activating TFs have more inputs

The results above suggest that auto-activation and auto-repression have very different functions. We therefore wondered whether in the known transcription regulatory network of *E. coli* the auto-activators and auto-repressors have different statistical properties.

Surprisingly, we found that auto-activators are more often regulated by other TFs than auto-repressors. According to the data in RegulonDB [2], 18 of the 25 auto-activating TFs in *E. coli* are regulated by at least one additional TF (72%) versus 30 out of 62 auto-repressing TFs (48%); this indeed suggests that auto-activators are more likely to have additional inputs (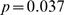). The difference becomes more convincing if we look at the total number of inputs for the two sets. The 25 auto-activators have, in total, 52 inputs (*i.e.*, an in-degree of 2.08 on average; the auto-regulation is not counted as an input) while the 62 auto-repressors have 50 inputs in total (0.81 on average). Evidently, auto-activators have significantly more inputs than auto-repressors (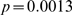).

Since auto-regulation can potentially have many functions and most of the auto-regulators are poorly characterized, we can only speculate about the origin of this difference. One possible explanation would be the following. If a common function of auto-activation is to shape response functions, as suggested by our analysis, then auto-activation should evolve preferentially for TFs that are regulated by one or more input TFs. In that case one would expect the average in-degree for auto-activators to be high. The same argument does not hold for auto-repression: our results suggest that auto-repression typically evolves for different reasons. Some of the functions of auto-repression suggested in the literature, such as its tendency to decrease intrinsic noise and to mitigate the effect of changes in the bacterial growth rates on gene expression, do not require additional input TFs. It is therefore not too surprising that for many auto-repressors (32 out of the 62) no input TF is known.

## Discussion

Our results shed new light on the use of auto-regulation. We described three situations in which auto-activation can be used to improve the response function of promoters. First, if auto-activation is conditional on the presence of other TFs, it can give rise to sensitive responses that otherwise require multiple cooperative binding sites of the input TF. Presumably, not all input TFs can bind cooperatively to multiple binding sites; in those cases conditional auto-activation can serve as an alternative. Secondly, auto-activation can strongly contribute to the sensitivity of the response of repression systems. Whenever sharp repression is required, auto-activation can have a selective advantage. Thirdly, we showed that auto-activation is also useful if a linearly decreasing response function is desired. Together, such mechanisms may help explain the large number of auto-activators present in *E. coli*.

Auto-repression never appeared in the simulation results if selection was based on the response function of the gates only. Most likely, the limited use of auto-repression in shaping response functions is due to its general tendency to decrease the fold-change and sensitivity of the response. A low fold-change or sensitivity can typically also be achieved without auto-repression by tuning both the promoter strength and the affinities of the TF binding sites. Nevertheless, we cannot exclude the possibility that auto-repression would show up in simulations selecting for response functions different from the ones presented here.

If the fitness function was altered to favor a fast response or suppression of intrinsic transcriptional noise, auto-repression did emerge. It has been suggested before that the function of negative auto-regulation is to regulate such dynamic properties [5], [9], [10]; our results support this view.

In retrospect, the emergence of auto-regulation is hardly surprising. The evolution of *cis*-regulatory regions can be perceived as adaptive curve fitting. Allowing for auto-regulation gives gene-regulatory systems additional degrees of freedom to optimize their performance, and it would perhaps be more surprising if this freedom were not exploited. We therefore expect that the conclusions based on the idealized gates studied in this work are also relevant for real biological systems requiring more complex response functions.

We have seen that in some cases the advantage of using auto-regulation is large (*e.g.* when sensitive repression is required) whereas in other cases there is only a small difference between the quality of the response function for designs with or without auto-regulation. This leads one to wonder whether in the latter case natural selection on the shape of the response would be large enough to evolve and maintain auto-regulation, in particular in the presence of noise. This is largely an open question; yet, the fact that some *E. coli* promoters contain a large number of TF binding sites many of which contribute only marginally to the expression (see for instance [29]) suggests that, at least in some *cis*-regulatory regions, natural selection is strong enough to fine-tune the response function in great detail.

The results presented are quite insensitive to the parameter values chosen. The value of 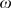 influences important properties such as the maximum fold change in activation systems, but as long as it is chosen within the biological range 10–100 the designs of the gates do not seem to depend qualitatively on the value chosen. To verify this, we performed simulations with 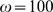 for AND, NAND, NOR and OR gates (without selection against noise or response speed) and found the results to be qualitatively the same as those presented. The value of 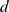 influences the spacing of binding sites within a module, but not the basic designs properties, as long as 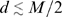 so that overlapping modules can be constructed that bind independently. The results are also insensitive to the length of the binding sites 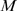 (we tested this with simulations for AND, NAND, NOR and OR gates with 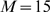) and the matrix elements of the binding energy matrix; essential is only that the evolutionary algorithm can tune the dissociation constants of the binding sites to a wide range of values (1–10000nM), as in reality. The length of the *cis*-regulatory region, 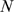, determines the maximum number of tandem binding sites that fit on the regulatory region; larger values of 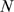 therefore ultimately lead to larger tandem arrays. However, since tandem arrays of five or more binding sites can form in the simulations, we believe that 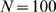 is large enough to accommodate typical *E. coli* promoters.

Even though in eukaryotes the mechanisms of gene regulation are generally different and various additional layers of regulation exist, recent work has shown that many basic principles of prokaryotic gene regulation—in particular the interplay between cooperative binding and competitive inhibition—are equally important in eukaryotes (see for instance [30] about repression and inhibition in yeast and [31] about enhancers in *Drosophila*). Auto-regulation is also widespread in eukaryotes [32]; therefore, our findings could also be relevant for gene regulation in eukaryotes.

As we mentioned, auto-activation is known to reduce the response speed in some situations and to increase the amplitude of fluctuations. Clearly, those issues may be problematic in some real-life situations. On the other hand, a slow response can be a positive feature as well if it is applied as a filter of high-frequency noise (a low-pass filter). Fluctuations may in some cases be beneficial or even necessary. For instance, when cells respond to a fluctuating environment via the strategy of stochastic switching, fluctuations are essential [33]. But even when cells cope with a fluctuating environment via the strategy of deterministic switching, fluctuations may be beneficial, since they can increase the population's growth rate when the response function is suboptimal [34]. Indeed, the fact that auto-activation is found so often in *E. coli* demonstrates that the associated reduction of the response speed and the amplification of fluctuations can apparently be circumvented, tolerated or put to use.

## Supporting Information

Text S1 Supplementary information. All supplementary information is contained in this document. It contains supplementary figures, detailed mathematical derivations, additional analyses, and numerical tests of approximations used in the main text.
(0.81 MB PDF)
