# Supplementary material for: Combinatorial Gene Regulation Using Auto-Regulation
Source: PLoS Comput Biol. 2010 Jun 10;6(6):e1000813. doi: 10.1371/journal.pcbi.1000813 (PMC2883594; doi:10.1371/journal.pcbi.1000813)
Supplement: Text S1 — Supplementary information. All supplementary information is contained in this document. It contains supplementary figures, detailed mathematical derivations, additional analyses, and numerical tests of approximations used in the main text. (0.81 MB PDF) [file pcbi.1000813.s001.pdf]

# Text S1: supplementary information for

# Combinatorial gene regulation using auto-regulation

Rutger Hermsen[1,*], Bas Ursem[2], Pieter Rein ten Wolde[2]
**1 Center for Biological Theoretical Physics, San Diego, CA, USA**
**2 FOM Institute AMOLF, Amsterdam, The Netherlands**
**∗ E-mail: hermsen@ctbp.ucsd.edu**

## 1 Minimal models

In the main text, several minimal models have been discussed to illustrate the mechanisms appearing in the simulation results. Here these models are defined more precisely.

In all descriptions below, the concentration of RNA polymerase–$\sigma$-complex in units of the promoter's dissociation constant is called $q_\mathrm{p}$. Transcription factors (TFs) binding cooperatively are assumed to interact with an energy $E_\mathrm{TF}$, resulting in a factor $\omega \equiv \exp(E_\mathrm{TF}) = 30$ in the Boltzmann weights of states in which both TFs are bound. Activators recruit RNAP through a similar cooperative interaction energy $E_\mathrm{R}$ and for simplicity we take $E_\mathrm{R} = E_\mathrm{TF}$.

We assume that the transcription rate of a promoter is proportional to the equilibrium fraction of time RNA polymerase (RNAP) is bound to the promoter, called $p_\mathrm{on}(c_1, c_2, c_3)$. The dynamics of the output concentration $c_3(t)$ are then assumed to obey:

$$\frac{\mathrm{d}c_3(t)}{\mathrm{d}t} = \alpha p_\mathrm{on}(c_1, c_2, c_3(t)) - \beta c_3(t). \tag{1}$$

(The dependence on $c_2$ is dropped if the gate has only one input.) The steady-state output concentration $c_3^*$ follows implicitly from

$$\alpha p_\mathrm{on}(c_1, c_2, c_3^*) - \beta c_3^* = 0. \tag{2}$$

In the absence of auto-regulation $p_\mathrm{on}$ does not depend on $c_3$ so that simply $c_3^* = (\alpha/\beta) p_\mathrm{on}(c_1, c_2)$. If $p_\mathrm{on}$ does depend on $c_3$ equation 2 has to be solved analytically or numerically.

The function $p_\mathrm{on}(c_1, c_2, c_3)$ can be expressed in terms of two partition sums: one over all states in which the promoter is occupied, $Z_\mathrm{on}$, and one sum over states in which the promoter is not occupied, $Z_\mathrm{off}$:

$$p_\mathrm{on}(c_1, c_2, c_3) = \frac{Z_\mathrm{on}(c_1, c_2, c_3)}{Z_\mathrm{on}(c_1, c_2, c_3) + Z_\mathrm{off}(c_1, c_2, c_3)}. \tag{3}$$

In the simulations these partition sums are calculated numerically using a dynamic programming algorithm [1], but for the simplified models they can be written out straightforwardly [2]. Below, $Z_\mathrm{on}$ and $Z_\mathrm{off}$ are specified for each of the models used; the response plot then follows from equation 2.[1]

[1] In all examples, equation 2 can be written as a polynomial equation; the order of the polynomial equation equals the number of auto-regulatory binding sites plus one.

## Conditional auto-activation

In Fig. 2C and D of the main text we compare the conventional mechanism of (cooperative) activation to conditional auto-activation.

The first system, activation by one binding site, is described by the following partition sums:

$$Z_{\text{on}}(c_1) = q_{\text{p}} \left(1 + \frac{\omega c_1}{k_{\text{A}}}\right), \tag{4a}$$

$$Z_{\text{off}}(c_1) = 1 + \frac{c_1}{k_{\text{A}}}, \tag{4b}$$

where $k_{\text{A}}$ is the dissociation constant of the binding site.

For the second system, cooperative activation by two binding sites, the following partition sums can be written down:

$$Z_{\text{on}}(c_1) = q_{\text{p}} \left(1 + \frac{c_1}{k_1} + \frac{\omega c_1}{k_2} + \frac{\omega^2 c_1^2}{k_1 k_2}\right), \tag{5a}$$

$$Z_{\text{off}}(c_1) = 1 + \frac{c_1}{k_1} + \frac{c_1}{k_2} + \frac{\omega c_1^2}{k_1 k_2}. \tag{5b}$$

Parameters $k_1$ and $k_2$ are the dissociation constants of the binding sites (from left to right in Fig. 2D of the main text).

The partition sums of the third system, with conditional auto-activation, are:

$$Z_{\text{on}}(c_1, c_3) = q_{\text{p}} \left(1 + \frac{c_3}{k^*} + \frac{\omega c_1}{k} + \omega^2 \frac{\omega^2 c_1 c_3}{k k^*}\right), \tag{6a}$$

$$Z_{\text{off}}(c_1, c_3) = 1 + \frac{c_3}{k^*} + \frac{c_1}{k} + \frac{\omega c_1 c_3}{k k^*}. \tag{6b}$$

Here $k$ and $k^*$ are the dissociation constants for the binding site of TF1 and TF3, respectively. Given these partition sums, equation 2 becomes a second order polynomial equation and can be solved analytically (the result is given in the main text).

Fig. 2D in the main text shows response plots for all three systems. In all cases we chose the parameter values that maximize the fitness function for ACT (activation) gates. The optimal values of the parameters are as follows. For the activator with one binding site: $k_{\text{A}} = 1.82\,\mu\text{M}$, $q_{\text{p}} = 0.179$. The sum of squared deviations for the optimal parameters is $M_{\text{RF}} = 0.81\,\mu\text{M}^2$. For cooperative activation by two binding sites: $k_1 = 10.8\,\mu\text{M}$, $k_2 = 2.50\,\mu\text{M}$, $q_{\text{p}} = 0.181$, and $M_{\text{RF}} = 0.61\,\mu\text{M}^2$. Conditional auto-activation leads to $k = 12.4\,\mu\text{M}$, $k^* = 2.68\,\mu\text{M}$, $q_{\text{p}} = 0.176$ and $M_{\text{RF}} = 0.66\,\mu\text{M}^2$.

## Sharper repression due to auto-activation

In Fig. 3C and D (main text) two systems are compared: cooperative repression and cooperative repression with additional auto-activation.

The partition sums of the first system are:

$$Z_{\text{on}}(c_1) = q_{\text{p}}, \tag{7a}$$

$$Z_{\text{off}}(c_1) = 1 + \frac{c_1}{k_1} + \frac{c_1}{k_2} + \frac{\omega c_1^2}{k_1 k_2}. \tag{7b}$$

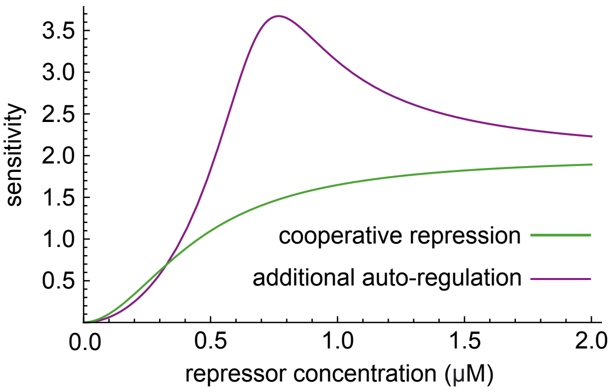


**Figure 1. Sensitivity $S(c_1)$ as a function of the input concentration $c_1$ for the plots in Fig. 3D of the main text.** The sensitivity of a response function $c_3^*(c_1)$ is defined as $S(c_1) \equiv |\mathrm{d}\log c_3^*/\mathrm{d}\log c_1| = |(c_1/c_3^*)\mathrm{d}c_3^*/\mathrm{d}c_1|$. Clearly, the sensitivity of the repression systems can be enhanced by adding an additional auto-regulation site.

In the second system, all states of the first system are possible, but on top of it the additional binding site for TF3 with dissociation constant $k^*$ can be bound. Because the repression module and the auto-activation binding site do not interact directly the partition sums factorize:

$$Z_{\mathrm{on}}(c_1, c_3) = q_{\mathrm{p}} \left(1 + \frac{\omega c_3}{k^*}\right), \tag{8a}$$

$$Z_{\mathrm{off}}(c_1, c_3) = \left(1 + \frac{c_1}{k_1} + \frac{c_1}{k_2} + \frac{\omega c_1^2}{k_1 k_2}\right) \left(1 + \frac{c_3}{k^*}\right). \tag{8b}$$

The plots shown have again been optimized using the fitness function for the IN (inhibit) gate. However, as it turns out, in both systems $q_{\mathrm{p}} \to \infty$ and $k_1, k_2 \to 0$ as the fitness is optimized. Clearly, this is unphysical. We therefore constrain the promoter strength by an arbitrary large number: $q_{\mathrm{p}} \leq 50$. The resulting parameters for the cooperative repression system are: $k_1 = k_2 = 344\,\mathrm{nM}$ and $q_{\mathrm{p}} = 50$, leading to $M_{\mathrm{RF}} = 0.34\,\mu\mathrm{M}^2$. The second system, with additional auto-actication, gives: $k_1 = k_2 = 134\,\mathrm{nM}$, $k^* = 1.75\,\mu\mathrm{M}$ and $q_{\mathrm{p}} = 50$, with $M_{\mathrm{RF}} = 0.15\,\mu\mathrm{M}^2$.

Note that the optimization returns identical values for $k_1$ and $k_2$; this maximizes the sensitivity of the repression. For that reason the analytical results in the main text (equation 9 and further) assume $k_1 = k_2$ from the start, which simplifies the calculation.

Fig. 1 shows the sensitivity function $S(c_1) \equiv \frac{c_1}{c_3^*}\frac{\mathrm{d}c_3^*}{\mathrm{d}c_1}$ for the optimized gates. Clearly, the sensitivity of the design with auto-activation reaches a much higher maximal sensitivity, as explained in the main text.

## Linear repression with auto-activation

The minimal model presented in Fig. 4A of the main text is identical to the second system in the previous subsection; the partition sums are given in equation 8. The usefulness of auto-activation in obtaining a more linear repression function can be understood as follows.

In the absence of auto-regulation $c_3^* = (\alpha/\beta)p_{\rm on}(c_1)$ so that

$$\frac{\mathrm{d}c_3^*}{\mathrm{d}c_1} = \frac{\alpha}{\beta}\frac{\mathrm{d}p_{\rm on}(c_1)}{\mathrm{d}c_1}. \tag{9}$$

To obtain linear repression this derivative should equal -1. For a cooperative repression system, $p_{\rm on}(c_1)$ has a sigmoidal shape and therefore $\frac{\mathrm{d}p_{\rm on}(c_1)}{\mathrm{d}c_1}$ is definitely not constant and equal to -1.

The equivalent of equation 9 for systems with auto-regulation can be derived from the relation $\alpha p_{\rm on}(c_1, c_2, c_3^*) = \beta c_3^*$:

$$\frac{\mathrm{d}c_3^*}{\mathrm{d}c_1} = \frac{\alpha}{\beta}\frac{\partial p_{\rm on}(c_1, c_3^*)}{\partial c_1} \Bigg/ \left(1 - \frac{\alpha}{\beta}\frac{\partial p_{\rm on}(c_1, c_3)}{\partial c_3}\bigg|_{c_3^*}\right). \tag{10}$$

This raises the hope that, by choosing the parameters correctly, the denominator in this equation may be tuned to mitigate some of the variation in the numerator.

We now illustrate this for the minimal model presented in Fig. 4A in the main text. The steady-state equation $\alpha p_{\rm on}(c_1, c_3^*) - \beta c_3^* = 0$ is quadratic in this case and can be solved exactly:

$$\frac{c_3^*(c_1)}{k^*} = \frac{-B(c_1) + \sqrt{B(c_1)^2 + 4A(c_1)C(c_1)}}{2A(c_1)}, \tag{11}$$

with

$$A(c_1) = 1 + \omega q_{\rm p} + \frac{c_1}{k_1} + \frac{c_1}{k_2} + \frac{\omega c_1^2}{k_1 k_2}, \tag{12}$$

$$B(c_1) = 1 - q_{\rm p}(\frac{\alpha\omega}{\beta k^*} - 1) + \frac{c_1}{k_1} + \frac{c_1}{k_2} + \frac{\omega c_1^2}{k_1 k_2}, \tag{13}$$

$$C(c_1) = \frac{\alpha q_{\rm p}}{\beta}. \tag{14}$$

It is straightforward to optimize this curve numerically with respect to the fitness function used in the main text, yielding the parameters $k^* = 731\,\mathrm{nM}, k_1 = 37.9\,\mathrm{nM}, k_2 = 7.17\,\mathrm{\mu M}, q_{\rm p} = 3.10$.

From the exact solution 11 it is not easy to understand why these parameters result in a rather straight curve in the interval $[0, 1\,\mathrm{\mu M}]$. More insight can be gained by examining equation 10. The right-hand side of this equation is determined by the partial derivatives of $p_{\rm on}$; in Fig. 2 we plot both derivatives as a function of $c_1$. If $c_1$ is large, the gate is completely inhibited ($c_3^*$ is low) and therefore the derivative $\partial p_{\rm on}(c_1, c_3^*)/\partial c_1$ must approach zero as well. At lower values of $c_1$ the derivative $\partial p_{\rm on}(c_1, c_3^*)/\partial c_1$ has a finite value. Equation 10 now shows that this variation in $\partial p_{\rm on}(c_1, c_3^*)/\partial c_1$ can be mitigated if the denominator correlates with the numerator. Fig. 2 shows that this is the case; it can be understood as follows. For any value of $c_1$, $p_{\rm on}$ is an increasing function of $c_3$ that saturates for high values of $c_3$. Therefore, for high values of $c_3^*$ the partial derivative $\partial p_{\rm on}(c_1, c_3)/\partial c_3$ must be small. This explains why 1-$(\alpha/\beta)\partial p_{\rm on}(c_1, c_3)/\partial c_3$ is almost 1 at $c_1 = 0$. At lower concentrations $c_3^*$ (*i.e.*, at higher values of $c_1$) $\partial p_{\rm on}(c_1, c_3)/\partial c_3$ increases. Since the absolute values of the denominator and the numerator correlate, their ratio is rather (but not quite) constant for different values of $c_1$.

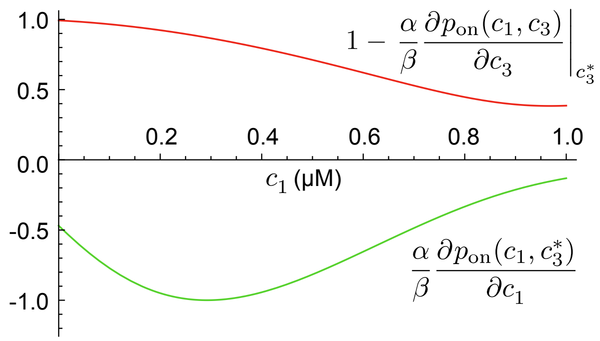


**Figure 2. Partial derivatives of $p_{\mathrm{on}}$ at steady state $c_3^*$, as a function of input concentration $c_1$.** Shown are the numerator and the denominator of equation10, for the minimal model depicted in Fig. 4A of the main text.

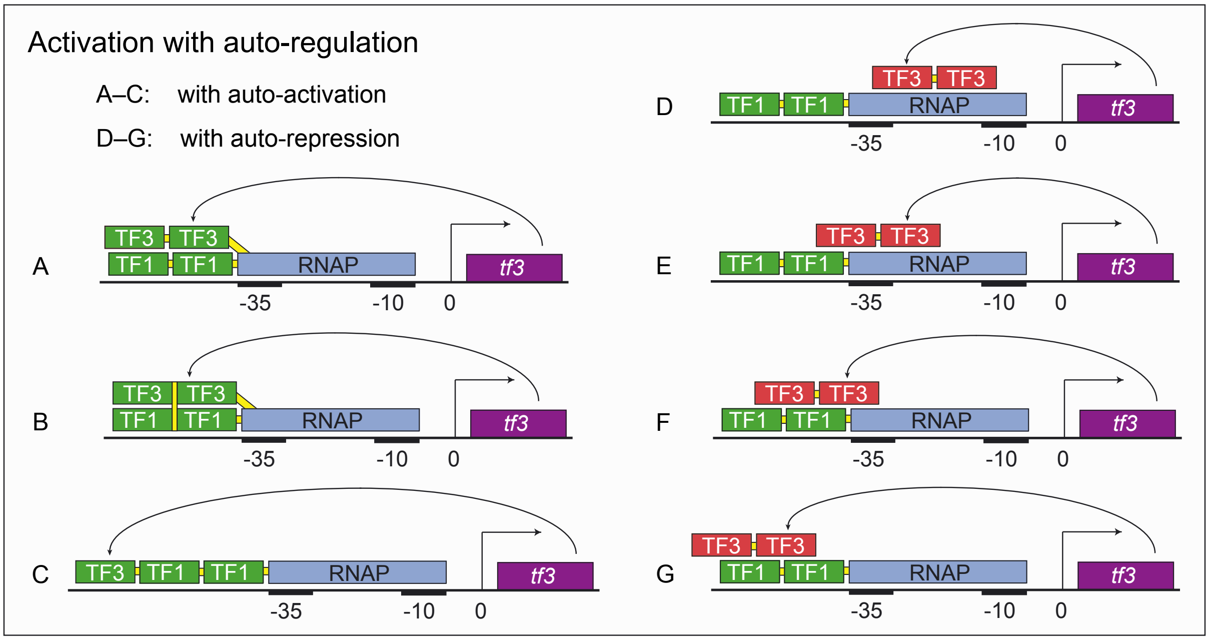


**Figure 3. Models of activation with auto-regulation**

## Linear activation with auto-regulation

In the simulation results, auto-regulation never occurred if we selected for a linear activation gate (LACT). To check if this was an artifact of the simulation scheme we studied the series of models depicted in Fig. 3. For all these models, a standard Nelder–Mead algorithm was used to find the binding affinities for the various binding sites that lead to the best LACT gate (as measured by the fitness function defined in the Method section of the main text). In all cases, the affinities of the auto-regulatory binding sites were zero in the optimized gates, confirming the results of the evolutionary algorithm. Each of the models is specified below.

We limited this analysis to designs containing modules of at most two binding sites. Each of the models has one activation module for the activator TF1 containing two binding sites; the dissociation constants of those sites are always called $k_1$ and $k_2$ (from left to right in Fig. 3). In addition, there are usually two binding sites for TF3, with dissociation constants $k_1^*$ and $k_2^*$. We consider several designs for auto-activation and for auto-repression. Auto-activation can be achieved through direct activation or through conditional auto-activation; both options are tested below. When auto-repression is combined with activation, the repression module can be placed in various locations, leading to different degrees of overlap with the activation module. We therefore consider a variety of placements.

**Model A** In this model two cooperative auto-activation sites are included that overlap with the operators for TF1. As a result, the modules for TF1 and TF3 compete for binding. The corresponding partition sums are:

$$Z_{\rm on}(c_1, c_3) = q_{\rm p}\left(1 + \frac{c_1}{k_1} + \frac{\omega c_1}{k_2} + \frac{c_3}{k_1^*} + \frac{\omega c_3}{k_2^*} + \frac{\omega^2 c_1^2}{k_1 k_2} + \frac{\omega^2 c_3^2}{k_1^* k_2^*} + \frac{\omega c_1 c_3}{k_1^* k_2}\right), \tag{15a}$$

$$Z_{\rm off}(c_1, c_3) = 1 + \frac{c_1}{k_1} + \frac{c_1}{k_2} + \frac{c_3}{k_1^*} + \frac{c_3}{k_2^*} + \frac{\omega c_1^2}{k_1 k_2} + \frac{\omega c_3^2}{k_1^* k_2^*} + \frac{c_1 c_3}{k_1^* k_2}. \tag{15b}$$

**Model B** This model is similar to model A, but now TF1 and TF3 can bind hetero-cooperatively to the sites that do not overlap (see Fig. 3), leading to additional states and factors in the partition sums:

$$Z_{\rm on}(c_1, c_3) = q_{\rm p}\left(1 + \frac{c_1}{k_1} + \frac{\omega c_1}{k_2} + \frac{c_3}{k_1^*} + \frac{\omega c_3}{k_2^*} + \frac{\omega^2 c_1^2}{k_1 k_2} + \frac{\omega^2 c_3^2}{k_1^* k_2^*} + \frac{\omega^2 c_1 c_3}{k_1 k_2^*} + \frac{\omega^2 c_1 c_3}{k_1^* k_2}\right), \tag{16a}$$

$$Z_{\rm off}(c_1, c_3) = 1 + \frac{c_1}{k_1} + \frac{c_1}{k_2} + \frac{c_3}{k_1^*} + \frac{c_3}{k_2^*} + \frac{\omega c_1^2}{k_1 k_2} + \frac{\omega c_3^2}{k_1^* k_2^*} + \frac{\omega c_1 c_3}{k_1 k_2^*} + \frac{\omega c_1 c_3}{k_1^* k_2}. \tag{16b}$$

**Model C** This model tests if conditional auto-activation could be used to produce linear response plots. There is only one binding site for TF3, with dissociation constant $k^*$.

$$Z_{\rm on}(c_1, c_3) = q_{\rm p}\left(1 + \frac{c_1}{k_1} + \frac{\omega c_1}{k_2} + \frac{c_3}{k^*} + \frac{\omega^2 c_1^2}{k_1 k_2} + \frac{\omega c_1 c_3}{k^* k_1} + \frac{\omega c_1 c_3}{k^* k_2} + \frac{\omega^3 c_1^2 c_3}{k_1 k_2 k^*}\right), \tag{17a}$$

$$Z_{\rm off}(c_1, c_3) = 1 + \frac{c_1}{k_1} + \frac{c_1}{k_2} + \frac{c_3}{k^*} + \frac{\omega c_1^2}{k_1 k_2} + \frac{\omega c_1 c_3}{k^* k_1} + \frac{c_1 c_3}{k^* k_2} + \frac{\omega^2 c_1^2 c_3}{k_1 k_2 k^*}. \tag{17b}$$

**Model D** Here activation is combined with auto-repression. The repressor sites are assumed not to overlap with the activator sites, and therefore $Z_{\text{off}}$ factorizes.

$$Z_{\text{on}}(c_1, c_3) = q_{\text{p}} \left( 1 + \frac{c_1}{k_1} + \frac{\omega c_1}{k_2} + \frac{\omega^2 c_1^2}{k_1 k_2} \right), \tag{18a}$$

$$Z_{\text{off}}(c_1, c_3) = \left( 1 + \frac{c_1}{k_1} + \frac{c_1}{k_2} + \frac{\omega c_1^2}{k_1 k_2} \right) \left( 1 + \frac{c_3}{k_1^*} + \frac{c_3}{k_2^*} + \frac{\omega c_3^2}{k_1^* k_2^*} \right). \tag{18b}$$

**Model E** This model is similar to model D, except that one of the auto-repressor sites overlaps with an activator site, eliminating several states in $Z_{\text{off}}$:

$$Z_{\text{on}}(c_1, c_3) = q_{\text{p}} \left( 1 + \frac{c_1}{k_1} + \frac{\omega c_1}{k_2} + \frac{\omega^2 c_1^2}{k_1 k_2} \right), \tag{19a}$$

$$Z_{\text{off}}(c_1, c_3) = 1 + \frac{c_1}{k_1} + \frac{c_1}{k_2} + \frac{c_3}{k_1^*} + \frac{c_3}{k_2^*} + \frac{\omega c_1^2}{k_1 k_2} + \frac{\omega c_3^2}{k_1^* k_2^*} + \frac{c_1 c_3}{k_1 k_1^*} + \frac{c_1 c_3}{k_1 k_2^*} + \frac{c_1 c_3}{k_2 k_2^*}. \tag{19b}$$

**Model F** In this model, the binding sites for TF3 both overlap with those of TF1; as a result, only one of the TF3 binding sites overlaps directly with the core promoter.

$$Z_{\text{on}}(c_1, c_3) = q_{\text{p}} \left( 1 + \frac{c_1}{k_1} + \frac{\omega c_1}{k_2} + \frac{c_3}{k_1^*} + \frac{\omega^2 c_1^2}{k_1 k_2} \right), \tag{20a}$$

$$Z_{\text{off}}(c_1, c_3) = 1 + \frac{c_1}{k_1} + \frac{c_1}{k_2} + \frac{c_3}{k_1^*} + \frac{c_3}{k_2^*} + \frac{\omega c_1^2}{k_1 k_2} + \frac{\omega c_3^2}{k_1^* k_2^*} + \frac{c_1 c_3}{k_1 k_2^*}. \tag{20b}$$

**Model G** In this last model, the binding sites for TF3 have shifted even further, so that the auto-regulation merely hinders the binding of the activator (anti-activation):

$$Z_{\text{on}}(c_1, c_3) = q_{\text{p}} \left( 1 + \frac{c_1}{k_1} + \frac{\omega c_1}{k_2} + \frac{c_3}{k_1^*} + \frac{c_3}{k_2^*} + \frac{\omega^2 c_1^2}{k_1 k_2} + \frac{\omega c_3^2}{k_1^* k_2^*} + \frac{\omega c_1 c_3}{k_1^* k_2} \right), \tag{21a}$$

$$Z_{\text{off}}(c_1, c_3) = 1 + \frac{c_1}{k_1} + \frac{c_1}{k_2} + \frac{c_3}{k_1^*} + \frac{c_3}{k_2^*} + \frac{\omega c_1^2}{k_1 k_2} + \frac{\omega c_3^2}{k_1^* k_2^*} + \frac{c_1 c_3}{k_1^* k_2}. \tag{21b}$$

In all models A to G, the affinities of the auto-regulatory binding sites were zero in the optimized gates, confirming the results of the evolutionary algorithm.

# 2 Computational footprints and deducing promoter designs

As explained in the main text, binding affinities are continuous variables and therefore there is no natural distinction between binding sites and non-binding sites. Nevertheless it is useful to evaluate which sites in the resulting designs are mainly responsible for the behavior of the gate.

A direct cut-off based on the binding equilibrium constants is not reliable because, in particular in the presence of cooperativity, weak binding sites can be very important. Therefore, in order to recognize TF binding sites we calculate occupancy profiles—computational "footprints".

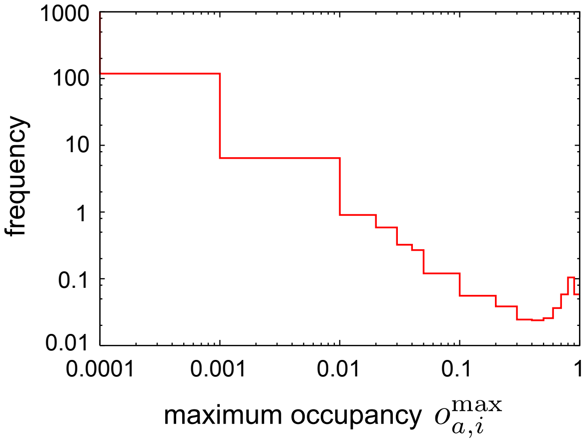


**Figure 4. Histogram of occupancies**

## Calculating occupancies

The occupancy of TF $a$ at site $i$ for input concentrations $(c_1, c_2)$ is calculated in the following way. First, we calculate the steady state value of $c_3$, called $c_3^*$, numerically. The occupancy can then be expressed as

$$o_{a,i} = \frac{Z(c_1, c_2, c_3^*|a \text{ bound to } i)}{Z_{\text{tot}}(c_1, c_2, c_3^*)} = 1 - \frac{Z(c_1, c_2, c_3^*|a \text{ not bound to } i)}{Z_{\text{tot}}(c_1, c_2, c_3^*)}. \tag{22}$$

Here $Z(c_1, c_2, c_3^*|a \text{ bound to } i)$ is the partition sum of all states in which $a$ is bound to $i$, and $Z(c_1, c_2, c_3^*|a \text{ not bound to } i)$ is the partition sum of all states in which $a$ is *not* bound to $i$. $Z_{\text{tot}}(c_1, c_2, c_3^*)$ is the total partition sum of the system.

The total partition $Z_{\text{tot}}(c_1, c_2, c_3)$ can easily be calculated with the recursive (dynamic programming) method described in Ref. [1]. To compute $Z(c_1, c_2, c_3^*|a \text{ not bound to } i)$ the exact same calculation is repeated, but now assuming that the dissociation constant describing the binding of $a$ to site $i$, called $k_{a,i}$, is infinite so that $c_a/k_{a,i} = 0$. This eliminates all states in which $a$ is bound to $i$ from the partition sum. Now, the right hand side of equation 22 can be evaluated.

## Defining binding sites

In order to separate important sites from unimportant ones we use a threshold based on the occupancy data. To determine a reasonable threshold value, we analyzed the distribution of occupancies in the simulation results. We define $o_{a,i}^{\max}$ to be the maximal occupancy of site $i$ by TF $a$ over the four conditions $(c_1, c_2) \in \{(0,0), (0, 1000\,\text{nM}), (1000\,\text{nM}, 0), (1000\,\text{nM}, 1000\,\text{nM})\}$. Figure 4 depicts a histogram of these occupancies for all TFs and all sites, using data gathered from the results of 200 simulations. Clearly, this histogram is bi-modal. The vast majority of the maximal occupancies $o_{a,i}^{\max}$ have negligible values. However, a second peak occurs around $o_{a,i}^{\max} = 0.9$; it is associated with binding sites that have evolved under selection pressure. Based on this histogram, we choose a rather stringent threshold for our initial estimate as to which

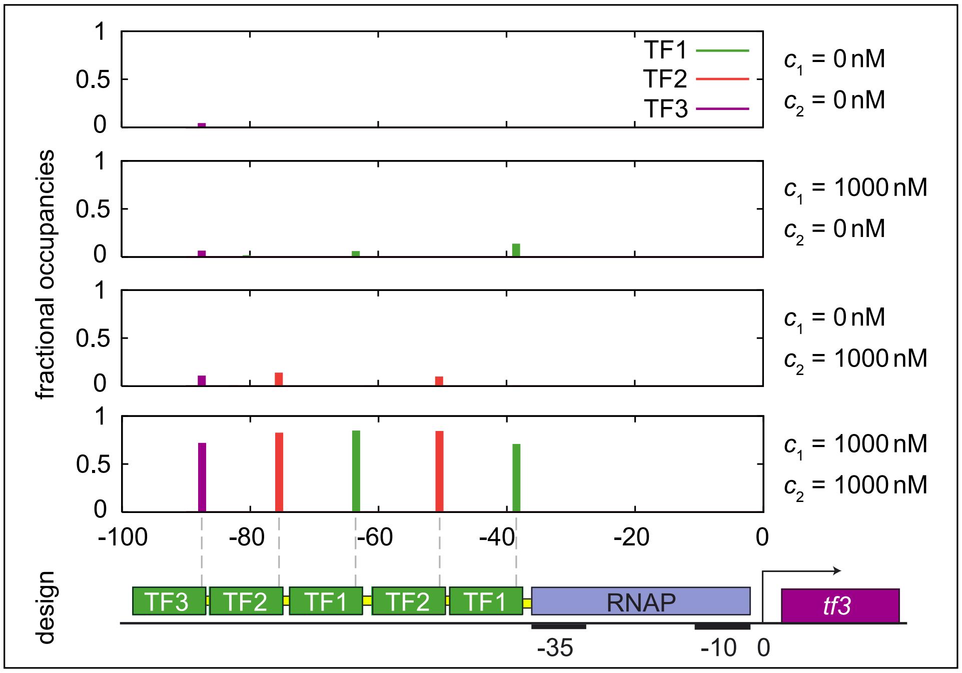


**Figure 5. From occupancy data to promoter design** The figure shows the fractional occupancies of all sites on the *cis*-regulatory region for an AND gate resulting from the simulations. The occupancies are calculated for four input conditions $(c_1, c_2)$, each shown in a separate plot. The occupancy of a site is indicated as a bar at the right-most base pair of the site.
It is clear that the occupancies of all sites are low except when both TF1 and TF2 are present at high concentrations (the fourth plot). There are five sites with a high occupancy (the affinities of all other sites are plotted as well, but remain invisible on this scale); these sites are therefore incorporated in the schematic promoter design below the plots. Indeed, simplified models including only these five sites accurately reproduce the response plot of this promoter.

binding sites have a function: $o_{a,i}^{\max} = 0.3$. The resulting simplified promoter designs usually accurately explain the responses of the corresponding full promoters. If the threshold is lowered, the resulting models more and more accurately approximate the behavior of the full promoters but the models become more and more complex.

### Deducing promoter designs from occupancy data

To illustrate how the promoter designs are deduced from the raw occupancy data, Fig. 5 shows the raw data for the AND gate presented in the main text (Fig. 2A).

## 3 Selecting against noise

In order to select against noise, we need to estimate the variance in the concentration of TF3 as a function of the input concentrations $(c_1, c_2)$. Because this calculation has to be repeated many times during one simulation (for 1000 generations, 200 organisms and 16 values of $(c_1, c_2)$

the number of repetitions becomes $3.2 \times 10^6$) a reasonable approximation is required that can be calculated rapidly. Below, we derive equations 20 and 21 from the main text and test their applicability.

### Deriving an approximate expression for the variance of $c_3$

In the main text, the following stochastic differential equation is introduced:

$$\frac{\mathrm{d}c_3(t)}{\mathrm{d}t} = \alpha\, p_{\mathrm{on}}(c_1, c_2, c_3(t)) - \beta\, c_3(t) + \xi(t). \tag{23}$$

Here $c_i$ is the concentration of TF $i$. The noise term $\xi(t)$ represents the fluctuations in the transcription, translation and degradation rates. The amplitude of the noise is given by:

$$\langle \xi(t')\xi(t)\rangle = ((\alpha/V)\, p_{\mathrm{on}}(c_1, c_2, c_3(t)) + \beta\, c_3(t)/V)\delta(t' - t). \tag{24}$$

The first term in the noise amplitude, $(\alpha/V)\, p_{\mathrm{on}}(c_1, c_2, c_3(t))$, describes the noise in the production of TF3 while the second term, $\beta\, c_3/V$, describes the stochasticity in the degradation of TF3. The form of this stochastic differential equation follows from the following considerations.

At a more fundamental level, the noise in the system can be treated by the following Master equation:

$$\begin{aligned}\frac{\partial P(c_3, t; c_1, c_2)}{\partial t} =& \alpha' p_{\mathrm{on}}\left(c_1, c_2, c_3 - \frac{1}{V}\right) P\left(c_3 - \frac{1}{V}, t; c_1, c_2\right) \\ &+ \beta'\left(c_3 + \frac{1}{V}\right) P\left(c_3 + \frac{1}{V}, t; c_1, c_2\right) \\ &- (\alpha' p_{\mathrm{on}}(c_1, c_2, c_3) + \beta' c_3)P(c_3, t; c_1, c_2).\end{aligned} \tag{25}$$

This equation describes the evolution of the probability distribution $P(c_3, t; c_1, c_2)$ (*i.e.*, the probability that the output concentration equals $c_3$ at time $t$ given the constant inputs $c_1$ and $c_2$) as a result of the stochastic processes of production and degradation. This Master equation can be approximated by a Fokker–Planck equation using the following Taylor expansions:

$$\begin{aligned}\alpha' p_{\mathrm{on}}\left(c_1, c_2, c_3 - \frac{1}{V}\right) P\left(c_3 - \frac{1}{V}, t; c_1, c_2\right) \approx& \alpha' p_{\mathrm{on}}(c_1, c_2, c_3)P(c_3, t; c_1, c_2) \\ &- \frac{1}{V}\frac{\partial}{\partial c_3}\alpha' p_{\mathrm{on}}(c_1, c_2, c_3)P(c_3, t; c_1, c_2), \\ &+ \frac{1}{2}\left(\frac{1}{V}\right)^2 \frac{\partial^2}{\partial c_3^2}\alpha' p_{\mathrm{on}}(c_1, c_2, c_3)P(c_3, t; c_1, c_2),\end{aligned} \tag{26}$$

$$\begin{aligned}\beta'\left(c_3 + \frac{1}{V}\right) P\left(c_3 + \frac{1}{V}, t; c_1, c_2\right) \approx& \beta' c_3 P(c_3, t; c_1, c_2) + \frac{1}{V}\frac{\partial}{\partial c_3}\beta' c_3 P(c_3, t; c_1, c_2) \\ &+ \frac{1}{2}\left(\frac{1}{V}\right)^2 \frac{\partial^2}{\partial c_3^2}\beta' c_3 P(c_3, t; c_1, c_2).\end{aligned} \tag{27}$$

Defining $\alpha \equiv \alpha'/V$ and $\beta \equiv \beta'/V$ we arrive at:

$$\frac{\partial P(c_3, t; c_1, c_2)}{\partial t} = -\frac{\partial}{\partial c_3} f(c_3; c_1, c_2) P(c_3, t; c_1, c_2) + \frac{\partial^2}{\partial c_3^2} g(c_3; c_1, c_2) P(c_3, t; c_1, c_2), \tag{28}$$

with

$$f(c_3; c_1, c_2) \equiv \alpha p_{\mathrm{on}}(c_1, c_2, c_3) - \beta c_3, \tag{29}$$
$$g(c_3; c_1, c_2) \equiv (\alpha p_{\mathrm{on}}(c_1, c_2, c_3) + \beta c_3)/2V. \tag{30}$$

In the Îto interpretation, this Fokker–Planck equation is mathematically equivalent to the stochastic differential equation 23 [3, 4]. This shows that the stochastic differential equation that we started with is an approximation of the Master equation above. (We test this approximation below.)

The steady state probability distribution $P^*(c_3; c_1, c_2)$ of the Fokker–Planck equation 28 is given by:

$$P^*(c_3; c_1, c_2) \propto \exp \int^{c_3} \left( f(c_3'; c_1, c_2) - \frac{\mathrm{d}g(c_3'; c_1, c_2)}{\mathrm{d}c_3'} \right) \Big/ g(c_3'; c_1; c_2)\, \mathrm{d}c_3'. \tag{31}$$

Because the function $p_{\mathrm{on}}(c_1, c_2, c_3)$ evolves during the simulation and is therefore generally unknown the integral on the right hand side cannot be performed analytically. However, anticipating that the distribution peaks close to $c_3^*$ (the equilibrium value of the deterministic equation 1) we can approximate the distribution by expanding $\log P^*(c_3; c_1, c_2)$ around $c_3 = c_3^*$:

$$\begin{aligned} \log P^*(c_3; c_1, c_2) \approx \text{const.} &+ (c_3 - c_3^*) \left[ \frac{f(c_3^*) - g'(c_3^*)}{g(c_3^*)} \right] \\ &+ \frac{1}{2}(c_3 - c_3^*)^2 \left[ \frac{g(c_3^*)(f'(c_3^*) - g''(c_3^*)) - (f(c_3^*) - g'(c_3^*))g'(c_3^*)}{g(c_3^*)^2} \right]. \end{aligned} \tag{32}$$

Note that we omitted the parameters $c_1$ and $c_2$ in the equations above for brevity. Using the definitions of $f(c_3)$ and $g(c_3)$ and the fact that $\alpha p_{\mathrm{on}}(c_1, c_2, c_3^*) = \beta c_3^*$ (by definition), we arrive at:

$$\log P^*(c_3; c_1, c_2) \approx \text{ const. } - a(c_3^*)(c_3 - c_3^*) - \frac{1}{2} b(c_3^*)(c_3 - c_3^*)^2, \tag{33}$$

with

$$a(c_3^*) \equiv \frac{(\alpha/\beta) p'_{\mathrm{on}}(c_1, c_2, c_3^*) + 1}{2c_3^*}, \tag{34}$$

$$b(c_3^*) \equiv V \frac{1 - (\alpha/\beta) p'_{\mathrm{on}}(c_1, c_2, c_3^*)}{c_3^*} - \left( \frac{(\alpha/\beta) p'_{\mathrm{on}}(c_1, c_2, c_3^*) + 1}{2c_3^*} \right)^2 + \frac{(\alpha/\beta) p''_{\mathrm{on}}(c_1, c_2, c_3^*)}{2c_3^*}. \tag{35}$$

The functions $p'_{\mathrm{on}}$ and $p''_{\mathrm{on}}$ are the first and second derivatives of $p_{\mathrm{on}}$ with respect to $c_3$. This approximated distribution is a Gaussian with variance

$$\sigma(c_3^*)^2 = \frac{1}{b(c_3^*)}. \tag{36}$$

This expression can easily be evaluated numerically.

We note that the volume $V$ of the cell enters explicitly in the expression for $b(c_3^*)$. Indeed, if the volume of the cell is increased *while the concentration* $c_3^*$ *is kept constant*, the mean copy number of TF3 in steady state is increased and therefore the concentration will fluctuate less. According to the deterministic equation 1 the steady state copy number of TF3 is $V\alpha p_{\rm on}/\beta < V\alpha/\beta$; we choose $V = 10^3\beta/\alpha$ so that the steady state copy numbers of TF3 stay within the range 1–$10^3$. (Since we use $\alpha/\beta = 1000\,\text{nM}$ this means that $V = 10^3/1000\,\text{nM} = 1.7\,\mu\text{m}^3$, which is indeed roughly the volume of one *E. coli* bacteria.)

## Testing the approximation

Of course, the approximation in equation 32 is not exact. We tested the quality of the approximation in the following manner.

In the case of auto-repression the dependence of $p_{\rm on}$ on $c_3$ is usually rather well described by a Hill-type function of the form:

$$p_{\rm on}(c_3) = \frac{Z_{\rm on}}{Z_{\rm on} + Z_{\rm off}} = \frac{q_{\rm p}}{q_{\rm p} + 1 + (c_3/k)^n}. \tag{37}$$

We therefore test the approximation for auto-repressors that obey this equation. For given values of $q_{\rm p}$ and $n$, the deterministic steady state concentration $c_3^*$ can be tuned by varying $k$. For the different values of $k$ we then calculate the variance of the steady state probability distribution using the approximation of equation 36. Next, we also calculate the mean and variance of $c_3$ exactly using equation 31; in order to do this, the integral on the right-hand side was performed numerically. Lastly we also calculate the mean and variance by solving the Master equation 25 numerically. The three results can then be compared.

In the case of auto-activation $p_{\rm on}$ is expected to have the following Hill-type form:

$$p_{\rm on}(c_3) = \frac{Z_{\rm on}}{Z_{\rm on} + Z_{\rm off}} = \frac{q_{\rm p}(1 + \omega(c_3/k)^n)}{q_{\rm p}(1 + \omega(c_3/k)^n) + 1 + (c_3/k)^n}. \tag{38}$$

Again, for given $q_{\rm p}$, $\omega$ and $n$, the deterministic steady state concentration $c_3^*$ can be tuned by varying $k$ and the corresponding variance can be calculated approximately using equation 36; subsequently we also use equation 31 and Master equation 25 to calculate the mean and variance according to the Fokker–Planck equation and the Master equation, respectively.

Fig. 6 show results of the tests. They show that the approximation is surprisingly accurate, even for low values of $c_3^*$. The mean and variance calculated with the approximation are practically indistinguishable from the exact Fokker–Planck or Master equation results. This justifies the use of the approximation in our heuristic fitness function.

## Limitations of the approximation

There are two conditions under which the approximation breaks down. The first condition is if $c_3^* \lesssim 1/V$, *i.e.*, if the equilibrium copy number of TF3 is $\lesssim 1$. Under these conditions the second term in the right-hand side of equation 35 becomes of the same order as the first term, so that $b(c_3^*)$ could become negative. In the simulations this happens in the initial phase of the

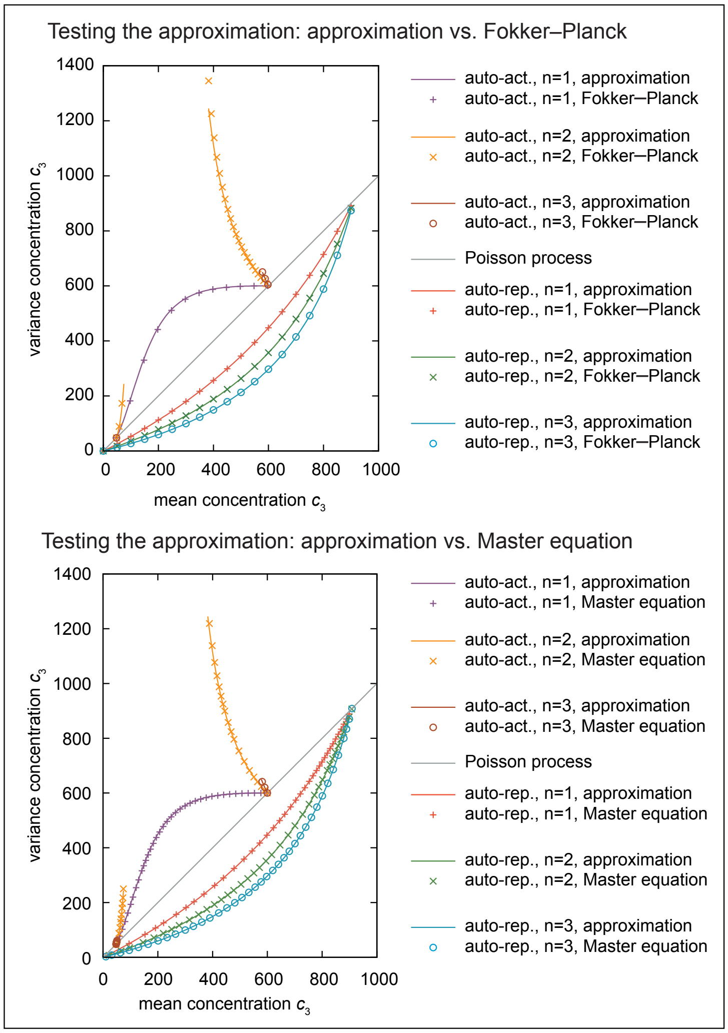


**Figure 6.** **Test of the approximate noise calculation** Both figures plot the variance of the concentration $c_3$ versus its mean, calculated in three different ways. The top plot compares the approximation of equation 36 with the exact solution equation 31 of the Fokker–Planck equation while the bottom plot compares the same approximation to numerical solutions of the Master equation 25. Results are shown for auto-activation and for auto-repression, and for Hill coefficients $n = 1, 2$ and 3. We use $\omega = 30$, $\alpha/\beta = 1000\,\text{nM}$ and $V = 10^3\beta/\alpha$, as in the simulations; auto-activators have $q_\text{p} = 0.05$ whereas auto-repressors have $q_\text{p} = 10$. The various mean concentrations $c_3$ are obtained by varying the dissociation constant $k$. Since in the simulations bi-stable systems are excluded, we only plot results for parameters for which the system is mono-stable (according to the deterministic equation 1); this explains why in case of auto-activation if $n > 1$ the plot breaks up in two disjointed branches. The results show that the approximation is surprisingly accurate; the three approaches are practically indistinguishable.

In a Poisson process, the variance is equal to the mean; this line is also shown. The fact that the Poisson process separates the data for the auto-activators from those for the auto-repressors illustrates that the Fano factor of auto-activators is larger than 1, while the Fano factor for auto-repressors is smaller than 1.

simulation, when the promoter is very weak. We heuristically solve this problem by imposing that if $c_3^* < 1/V$ the standard deviation becomes $\sigma = 2/\sqrt{3}V$; this is the value predicted by equation 35 if $c_3^* = 1/V$ and $\alpha p'_{\mathrm{on}}/\beta \ll 1$. This heuristic fix should not significantly affect the simulation results since the total value of the noise measure is dominated by regions of the response function where $c_3^*$ is large.

The second condition under which the approximation fails is when $\alpha p'_{\mathrm{on}}(c_3^*)/\beta$ approaches 1. Under these conditions the noise becomes very large; indeed, the deterministic steady state solution becomes unstable (bifurcates) at $\alpha p'_{\mathrm{on}}(c_3^*)/\beta = 1$. Since we select against noise, the designs naturally stay away from this high-noise region of parameter space. Nevertheless, deleterious mutants in the population can occasionally enter this region. In this case we assign them the high standard deviation $\sigma = 1000\,\mathrm{nM}$; selection subsequently removes them from the population.

### OR gates under selection against noise

Figure 7 illustrates how selection against noise affects OR gates. In general there is a trade-off between the quality of the response plot and the amount of noise. This trade-off also determines whether auto-repression is beneficial. If noise is not under selection, auto-repression is not observed, but under moderate selection pressure against noise ($\gamma_{\mathrm{N}}^{\mathrm{max}} = 10$) the gates always do develop auto-repression (with feedback measure $M_{\mathrm{FB}} > 5.6 \times 10^4\,\mathrm{nM}^2$).

## 4 Statistical tests

In the main text, the results of two statistical tests are mentioned. Below we provide the details of the calculations.

### Auto-activators are more likely to have additional inputs

According to the data in RegulonDB [5], 18 of the 25 auto-activating TFs in *E. coli* have at least one additional input (72%) versus 30 out of 62 auto-repressing TFs (48%); this suggests that auto-activators are more likely to have additional inputs. The probability that out of $n = 25 + 62 = 87$ auto-regulating TFs, of which $r = 30 + 18 = 48$ have additional inputs, a random sample of size $l = 25$ contains $m = 18$ or more TFs with additional inputs equals

$$\sum_{i=m}^{l} \binom{r}{i}\binom{n-r}{l-i} \Big/ \binom{n}{l} = 0.037. \tag{39}$$

### Auto-activators have, on average, more inputs

The 25 auto-activators have, in total, 52 inputs (*i.e.*, 2.08 on average) while the 62 auto-repressors have 50 inputs in total (0.81 on average). To test if this difference is significant, we took the actual data and randomly permuted the *signs* of the auto-regulation $10^7$ times. Then, we counted how often in this set of permutations the 25 auto-activators had a total number of inputs equal to or greater than 52. As this happened 12966 times, $p = 0.0013$. We therefore conclude that auto-activators have significantly more inputs than auto-repressors.

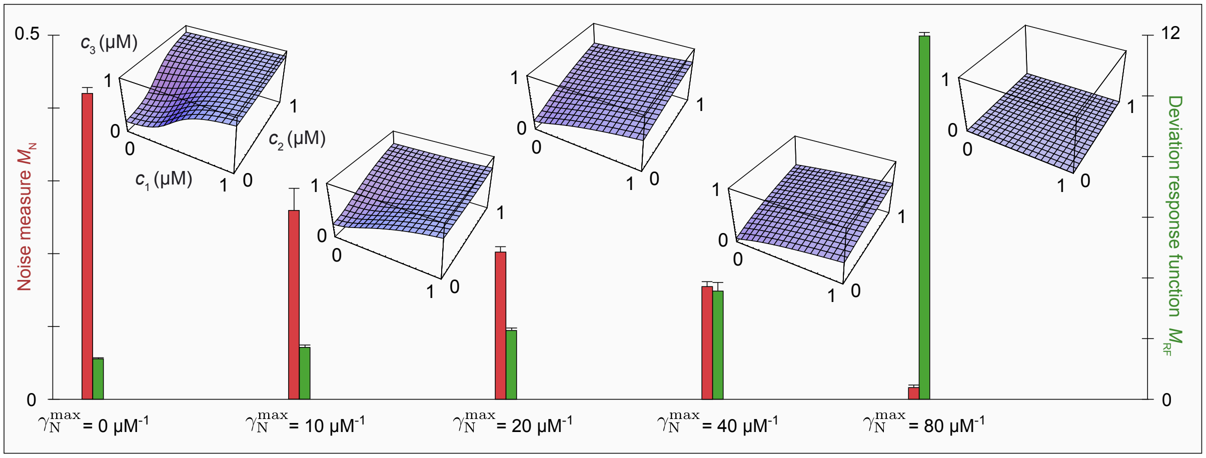


**Figure 7. OR gates under increasing selection against noise.** We carried out simulations with varying levels of selection against noise: $\gamma_N^{max} = 0\,\mu M^{-1}, 10\,\mu M^{-1}, 20\,\mu M^{-1}, 40\,\mu M^{-1}$ or $80\,\mu M^{-1}$. The figure shows representative response plots of OR gates that evolved under each of these conditions. Each simulation was repeated 20 times with different initial conditions; the plotted values of the noise measure $M_N$ (in units of μM) and the response quality measure $M_{RF}$ (in μM$^2$) are averages over these repeats (the standard deviations are also shown). Clearly, the noise decreases if the selection strength against noise is increased. But this decrease comes at a cost: the quality of the response function decreases (remember that both $M_N$ and $M_{RF}$ are *low* when the performance is good).
In all cases except $\gamma_N^{max} = 0$, all resulting gates use auto-repression to lower the noise. As a result, the fold-changes are reduced. Also, since the noise tends to be lower at lower values of $c_3$ (*c.f.* equation 36) the total expression level is decreased gradually as $\gamma_N^{max}$ increases until at $\gamma_N^{max} = 80\,\mu M^{-1}$, the response has disappeared altogether.

# 5 Linear repression

## References

1. Hermsen R, Tans S, ten Wolde PR (2006) Transcriptional regulation by competing transcription factor modules. PLoS Computational Biology 2: e164.

2. Bintu L, Buchler NE, Garcia HG, Gerland U, Hwa T, et al. (2005) Transcription regulation by the numbers 1: Models. Curr Opin Gen & Dev 15: 116–124.

3. Gardiner CW (2004) Handbook of stochastic methods for physics, chemistry, and the natural sciences. Berlin: Springer-Verlag, 3rd ed edition.

4. van Kampen NG (1992) Stochastic Processes in Physics and Chemistry. Amsterdam: North-Holland.

5. Salgado H, Santos-Zavaleta A, Gama-Castro S, Millan-Zarate D, Diaz-Peredo E, et al. (2001) RegulonDB (version 3.2): transcriptional regulation and operon organization in *Escherichia coli* k-12. Nucl Acids Res 29: 72–74.
